# Sternum-worn vibrotactile device in Parkinson’s disease: a randomised, double-blind, placebo-controlled pilot trial

**DOI:** 10.1038/s41531-026-01448-y

**Published:** 2026-07-03

**Authors:** Viktoria Azoidou, Essa Bhadra, Ellen Camboe, Kamalesh C. Dey, Alexandra Zirra, Kira Rowsell, Corrine Quah, Caroline Budu, Thomas Boyle, David Gallagher, Jonathan P. Bestwick, Alastair J. Noyce, Cristina Simonet

**Affiliations:** 1 Centre for Preventive Neurology, Wolfson Institute of Population Health, Queen Mary University of London, London, UK; 2 Department of Neurology, Royal London Hospital, Barts Health, London, UK; 3 Neurology Department, Homerton Healthcare NHS Foundation Trust, London, UK; 4 Older Person’s Services & GIM Special Interest in Parkinson’s Disease, Royal London Hospital, London, UK

**Keywords:** Diseases, Health care, Medical research, Neurology, Neuroscience

## Abstract

Effective, non-invasive treatments for motor and non-motor symptoms in Parkinson’s disease (PD) remain limited, and wearable vibrotactile stimulation may help by enhancing sensorimotor integration. This multi-centre, double-blind, pilot randomised controlled trial evaluated the usability, safety and tolerability of the CUE1+ sternum-worn vibrotactile device, with exploratory clinical outcomes compared with a sham device. Fifty adults with idiopathic PD, stable on antiparkinsonian medication, were randomised to active CUE1+ (*n* = 25) or sham (*n* = 25) for 12-weeks (8 h/day), with ON-medication assessments at baseline and week-13. Four participants (8%) discontinued; compliance exceeded 95% in both groups, and 2 (4.2%) experienced mild, transient skin irritation. Exploratory between-group differences favoured active stimulation on MDS-UPDRS Part III (−11.1 points; 95%CI −17.3 to −4.9) and the PDQ-39 Summary Index (−7.6 points; 95%CI −12.3 to −3.0). CUE1+ was usable, safe, and well tolerated. The exploratory clinical signals do not establish efficacy and require confirmation in adequately powered trials using an active vibrotactile sham. Trial registration: ClinicalTrials.gov, NCT06174948. Registered 13 February 2024.

## Introduction

Parkinson’s disease (PD) affects more than 12 million people worldwide and imposes a rising burden on patients, caregivers, and healthcare systems^1–3^. It is characterised by cardinal motor signs including bradykinesia, rigidity, and rest tremor, alongside axial features such as freezing of gait (FOG) and postural instability^2,4,5^. Despite advances in pharmacological and surgical approaches, motor and non-motor symptoms remain incompletely controlled, particularly in advanced stages^6,7^.

Dopaminergic therapies, especially levodopa, remain the gold standard but associated with fluctuations, dyskinesias, and reduced efficacy for axial symptoms over time^6,8,9^. Device-aided therapies, including deep brain stimulation (DBS), have demonstrated sustained improvements in selected patients, but are invasive, costly, and not suitable for all^7,10,11^. Previous studies have highlighted the role of DBS in modulating pathological basal ganglia activity, particularly abnormal beta-wave synchronisation, and its limitations in addressing axial symptoms^10,12^. At the same time, non-motor symptoms (e.g., sleep disturbance, depression, autonomic dysfunction) substantially affect quality of life (QoL) and are often inadequately addressed by medication or DBS^5,13,14^. These multidimensional challenges highlight the need for safe, accessible, non-invasive adjunctive therapies.

One promising avenue is sensory cueing and vibrotactile stimulation. External rhythmic cues (auditory, visual, tactile) can recruit cerebellar-thalamo-cortical and premotor-supplementary motor area (SMA) networks, compensating for impaired basal ganglia timing mechanisms^15–18^. Tactile/vibrotactile cues may modulate beta-synchronised activity within basal ganglia-cortical loops, facilitating initiation and continuation of movement^16,19,20^. Cueing has shown benefits for freezing of gait, bradykinesia, and gait variability in both experimental and real-world settings^17,18,21,22^. However, systematic reviews highlight important heterogeneity, small sample sizes, and frequent lack of blinding in existing studies of vibrotactile cueing^19,23^. Currently, high-quality, randomised, blinded trials with pragmatic endpoints are needed to determine real-world effectiveness^2,3,24^.

The CUE1+ device is a sternum-worn, non-invasive vibrotactile stimulator designed to provide rhythmic tactile cues during daily life in people with PD. In an earlier feasibility study, we showed high adherence and promising signals of benefit^24^. More recently, the CUE1 device has also demonstrated usability, safety, and tolerability in primary orthostatic tremor, with improvements in mobility, stance, and fatigue over nine weeks^25^, supporting the broader applicability of vibrotactile cueing across related movement disorders. Building on this, we conducted a double-blind randomised controlled trial (RCT) to assess usability and safety/tolerability of CUE1 + , with exploratory assessment of motor, gait, and patient-reported outcomes. We hypothesised that CUE1+ would be feasible to use at home, well tolerated, and associated with exploratory signals of motor benefit compared with sham.

## Results

### Recruitment and participants

**Fig. 1:**
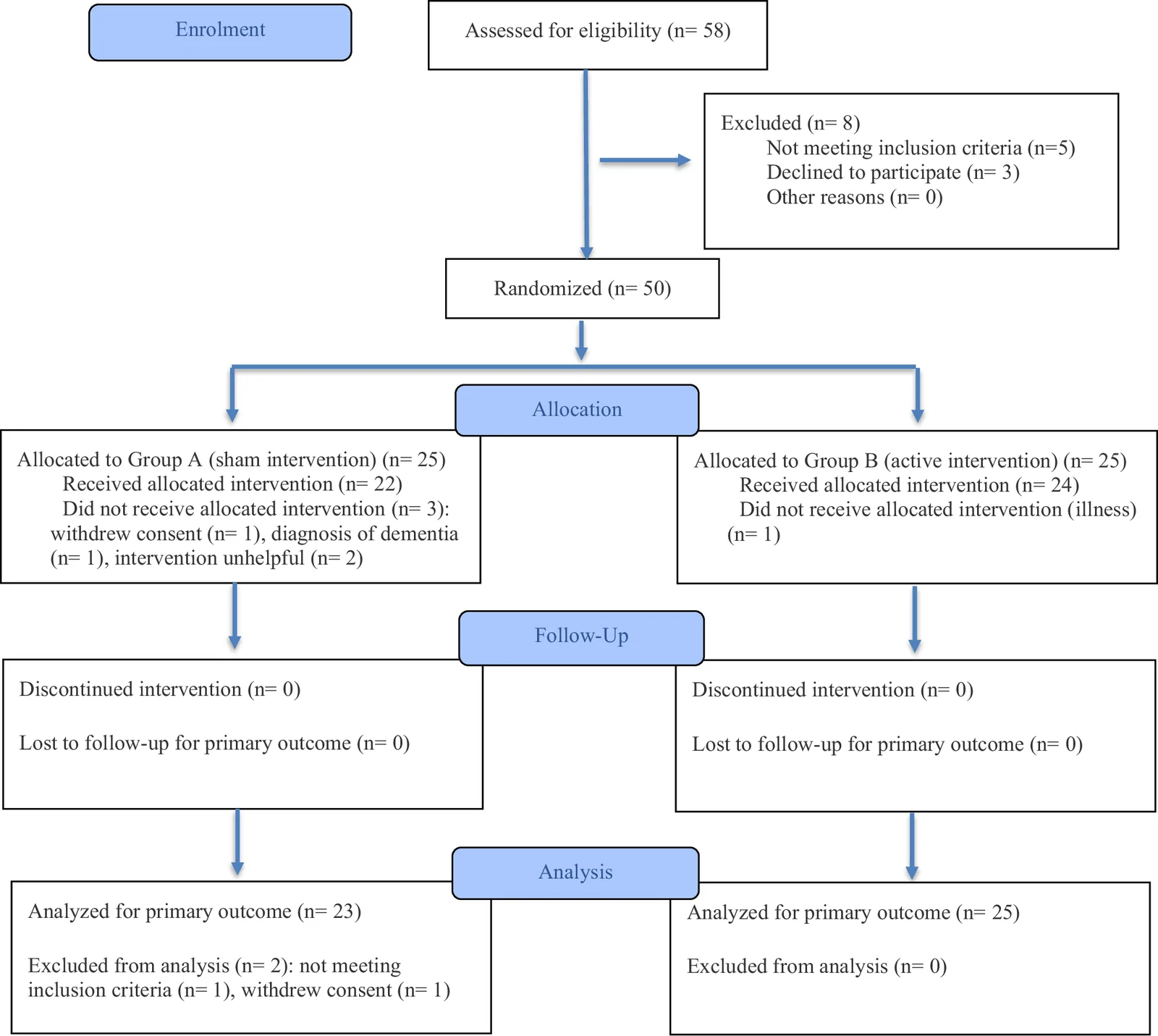
CONSORT 2025 flow diagram. Flow diagram showing participant screening, randomisation, allocation to sham or active device groups, follow-up, and inclusion in the intention-to-treat analysis in accordance with CONSORT 2025 guidelines.

Between September 1, 2024, and February 28, 2025, a total of 58 individuals were screened for eligibility: 13 (22.4%) at Homerton University Hospital and 45 (77.6%) at Barts Health NHS Trust. Of those screened, 5 (8.6%) did not meet inclusion criteria, and 3 (5.2%) declined participation due to personal reasons (Fig. 1). The remaining 50 were randomly assigned to the control/sham group or the intervention/active device group. Of those randomised, 7 (14.0%) were recruited from Homerton University Hospital and 43 (86.0%) from Barts Health NHS Trust. Baseline demographic and clinical characteristics are shown in Table 1. Seven (30%) of participants in the sham group and 11 (44%) in the active group were from non-white ethnic origin. Recruitment rate was 86%, consistent with our previous clinical studies^24,25^. Four participants (8.0%) discontinued the trial, 3 (6.3%) from the sham group and 1 (2.0%) from the active group.^26^

**Table 1:** Baseline characteristics of the intention-to-treat population

Characteristic	Sham device group (*n* = 23)	Active device group (*n* = 25)
Age, years	70.0 (65.0–75.5)	68.0 (62.0–75.0)
Sex, male	14 (60.9%)	13 (52.0%)
Sex, female	9 (39.1%)	12 (48.0%)
Ethnic origin, White	16 (69.6%)	14 (56.0%)
Ethnic origin, Black	0 (0.0%)	3 (12.0%)
Ethnic origin, Asian	7 (30.4%)	8 (32.0%)
Symptom duration, years	5.0 (2.5–7.0)	5.0 (2.0–8.0)
Hoehn and Yahr stage	3.0 (2.5–3.0)	3.0 (2.0–3.0)
Most affected side, right	14 (56.0%)	11 (44.0%)
Most affected side, left	10 (43.5%)	13 (56.5%)
Levodopa equivalent daily dose, mg/day	400 (300–400)	400 (300–500)
Montreal Cognitive Assessment score	26.0 (24.5–28.0)	25.0 (23.0–27.0)
MDS-UPDRS Part III score	44.0 (10.4)	43.4 (15.2)

Data are presented as *n* (%), median (IQR), or mean (SD), as appropriate. LEDD levodopa equivalent daily dose; MDS-UPDRS Movement Disorder Society-Unified Parkinson’s Disease Rating Scale.

### Safety and tolerability

The intervention was well tolerated across both groups, with minimal adverse events. Mild, transient skin irritation at the adhesive patch site occurred in two of 48 participants (4.2%; *n* = 48), one in each group, and resolved without treatment. One participant (2.1%) in the active group reported dizziness during use at the pre-set vibration intensity; however, this coincided with generalised dizziness unrelated to the device, and the participant withdrew due to an intercurrent illness. After the trial, they resumed use of the CUE1+ independently and increased vibration intensity from 50% to 70% via the CUE App (not used during the trial), without recurrence of dizziness. No participants reported difficulty with patch application or removal. A single technical malfunction occurred in the active group and was promptly resolved by an unblinded researcher (CS) in coordination with Charco Neurotech Ltd. A replacement device was delivered within days, and no further technical issues were reported. Overall, the intervention demonstrated a favourable safety and usability profile.

### Usability and compliance

Participant-level usability and compliance are shown in Table 2. There was no notable between-group difference. On the post-study satisfaction questionnaire, participants in the active group rated the CUE1+ as more helpful in managing their symptoms compared with those in the sham group [−1.1(−1.7, −0.5), *p* = 0.001], expressed a higher likelihood of continuing device use beyond the trial period [1.0(0.5, 1.0), *p* = 0.004], but showed no between-group difference in willingness to recommend the device to others with PD [0.0(0.0, 1.0), *p* = 0.34] (Supplementary Table 5).

**Table 2:** Participant-level usability and compliance to intervention

Measure	Sham device group (*n* = 23)	Active device group (*n* = 25)
Self-reported daily device use (≥8 h/day)	22 (95.7%)	25 (100.0%)
Self-reported inconsistent use	1 (4.3%)	0 (0.0%)
Self-rated compliance score 10 (fully compliant)	21 (91.3%)	21 (84.0%)
Self-rated compliance score 7–9 (high compliance)	1 (4.3%)	4 (16.0%)
Self-rated compliance score 4–6 (moderate compliance)	1 (4.3%)	0 (0.0%)

Data are presented as *n* (%). Daily use was defined as use of the device for at least 8 h per day on most days during the study period. Compliance was additionally assessed using a self-rated visual analogue scale from 0 to 10, where higher scores indicate greater perceived compliance.

### Motor function and QoL

**Fig. 2:**
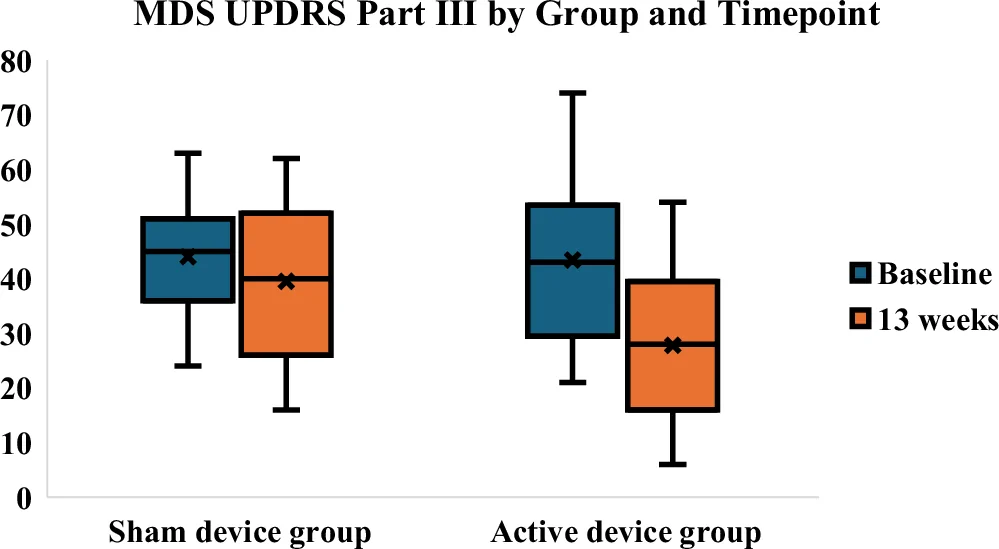
MDS-UPDRS Part III scores by group and timepoint. Box plots showing MDS-UPDRS Part III motor examination scores at baseline and week 13 for the sham device group and the active device group. Boxes represent the interquartile range with the median indicated; whiskers show minimum and maximum values. The sham device group demonstrated a mean change of −4.5 points from baseline to week 13 (95% confidence interval −8.7 to −0.4; *p* = 0.04), while the active device group showed a mean change of −15.6 points (95% confidence interval −20.3 to −10.9; *p* < 0.001). The between-group difference in change was 11.1 points (95% confidence interval 4.9 to 17.3; *p* = 0.002). Following Bonferroni correction for multiple comparisons, the threshold for statistical significance was adjusted from *p* < 0.05 to *p* < 0.004.

At baseline, there were no significant between-group differences for secondary exploratory outcomes (Supplementary Tables 6 and 7). At follow-up, two outcomes met the pre-specified Bonferroni-corrected significance threshold of *p* < 0.004: MDS-UPDRS Part III (between-group difference 11.1 points, 95%CI 4.9 to 17.3; *p* = 0.002; Fig. 2) and PDQ-39 Summary Index (SI) (between-group difference −7.6 points, 95%CI -12.3 to −3.0; *p* = 0.003; Tables 3–4). In an ANCOVA adjusting for baseline MDS-UPDRS Part III, baseline age, and baseline LEDD, the adjusted mean follow-up score was 27.9 in the active group and 39.4 in the sham group, yielding an adjusted between-group difference of −11.4 points (95%CI −17.9 to −5.0; *p* < 0.001) (Supplementary Table 8). This ANCOVA-adjusted estimate did not meet the Bonferroni-corrected threshold of *p* < 0.004 and is reported as a supportive sensitivity analysis only. Neither baseline age (*β* = −0.05, *p* = 0.814) nor baseline LEDD (*β* = 0.00, *p* = 0.985) was an independent significant predictor of follow-up motor score, consistent with the balance between groups on both variables (Table 1). Improvements were also observed in disease-specific QoL. The unadjusted between-group difference in PDQ-39 Summary Index at follow-up was −7.6 points (95% CI −12.3 to −3.0; *p* = 0.003). In a supportive ANCOVA adjusting for baseline PDQ-39 SI, age, and LEDD, the adjusted between-group difference was −7.2 points (95% CI −14.5 to 0.1; *p* = 0.05), consistent in the same direction after covariate adjustment (Supplementary Table 9). Exploratory, greater changes were seen in bradykinesia-related items than axial items, with smaller and more variable effects in tremor and rigidity (Supplementary Tables 10-13).

**Table 3:** Between group difference in outcomes at follow up

Outcome	Domain	Sham device (*n* = 23)	Active device (*n* = 25)	Between-group difference (95% CI)	*P* value
MDS-UPDRS	Part I	13.0 (10.5–17.5)	15.0 (11.0–24.0)	2.0 (–3.0 to 6.0)	0.44
	Part II	15.0 (7.0–22.5)	12.0 (9.0–19.0)	–2.0 (–7.0 to 4.0)	0.54
	Part IV	8.0 (5.0–11.0)	5.0 (4.0–9.0)	–2.0 (–5.0 to 0.0)	0.05
Functional Gait Assessment	Total	17.6 (4.2)	19.0 (16.0–22.0)	1.5 (–0.8 to 3.8)	0.18
Timed Up and Go	Single task	10.7 (3.6)	9.0 (8.4–10.8)	–0.3 (–2.2 to 1.4)	0.73
	Dual task	13.3 (9.7–18.5)	12.1 (10.6–17.5)	0.3 (–3.2 to 3.2)	0.92
BRAIN tap test	Most affected side	38.0 (36.0–47.0)	47.0 (40.0–55.0)	4.1 (–1.6 to 10.2)	0.62
Digital Finger Tapping	Most affected side	63.5 (49.0–80.0)	76.0 (54.0–87.0)	–12.5 (–27.5 to 14.0)	0.32
Activities-specific Balance Confidence	Total	62.5 (24.8)	56.3 (47.5–86.3)	3.8 (–4.4 to 17.5)	0.09
Pittsburgh Sleep Quality Index	Total	10.2 (4.9)	8.0 (5.0–13.0)	–1.0 (–2.0 to 0.0)	0.06
Parkinson’s Disease Questionnaire-39	Summary Index	27.8 (11.9–42.0)	29.1 (17.7–40.4)	–7.6 (–12.3 to –3.0)	0.003
Hospital Anxiety and Depression Scale	Anxiety	5.0 (3.0–9.0)	10.0 (7.0–12.0)	3.0 (0.0–5.0)	0.04
	Depression	6.0 (4.0–9.5)	8.0 (5.0–11.0)	0.0 (–2.0 to 1.0)	0.45
Fatigue Severity Scale	Total	40.0 (19.5–46.0)	36.0 (32.0–44.0)	4.0 (–17.0 to 10.0)	0.52

Data are median (IQR) or mean (SD), as appropriate, representing absolute scores at week-13 follow-up. Between-group differences are expressed as estimated mean or median differences with 95% confidence intervals. No significant between-group differences were present at baseline for any of these outcomes (Supplementary Tables 6 and 7). Between-group differences at follow-up are therefore interpretable as equivalent to between-group differences in change from baseline.

*CI* confidence interval, *MDS-UPDRS* Movement Disorder Society-Unified Parkinson’s Disease Rating Scale.

**Table 4:** Proportion of participants achieving minimal clinically important difference at follow-up

Outcome	Sham device (*n* = 23)	Active device (*n* = 25)
MDS-UPDRS Part I	7 (30.4%)	17 (68.0%)
MDS-UPDRS Part II	5 (21.7%)	13 (52.0%)
MDS-UPDRS Part IV	7 (30.4%)	21 (84.0%)
PDQ-39 Summary Index	4 (17.4%)	14 (56.0%)
PDQ-39 Mobility	5 (21.7%)	15 (60.0%)
PDQ-39 Activities of daily living	8 (34.8%)	16 (64.0%)
PDQ-39 Emotional wellbeing	5 (20.0%)	12 (48.0%)
PDQ-39 Stigma	6 (26.1%)	13 (52.0%)
PDQ-39 Social support	0 (0.0%)	4 (16.0%)
PDQ-39 Cognition	9 (39.1%)	16 (64.0%)
PDQ-39 Communication	4 (17.4%)	13 (52.0%)
PDQ-39 Bodily discomfort	10 (43.5%)	8 (32.0%)

Minimal clinically important differences were predefined for outcomes where established in Parkinson’s disease. Where MCID values are not established in Parkinson’s disease, results are presented descriptively; PDQ-39 Parkinsons’s Disease Questionnaire-39.

### Other secondary outcomes

No statistically significant between-group difference was observed for the remaining 13 secondary outcomes (Tables 3–4) including MDS-UPDRS Parts I (p = 0.44), II (*p* = 0.54), or IV (*p* = 0.05); FGA (*p* = 0.18); TUG single-task (*p* = 0.73) or dual-task (*p* = 0.92); BRAIN-KS (*p* = 0.62); DFT-KS (*p* = 0.32); balance confidence (ABC; *p* = 0.09); sleep quality (PSQI; *p* = 0.06); fatigue (FSS; *p* = 0.52); HADS depression (*p* = 0.45); or HADS anxiety. None met the Bonferroni-corrected threshold of *p* < 0.004. PGIC ‘Change Since Beginning of Care’ showed a numerical difference favouring the active group [median 4.0 (IQR 2.0–5.0)] over the sham group [2.0 (IQR 1.0–3.5); *p* = 0.05] but also did not meet the corrected threshold. Although the active group demonstrated within-group improvements across several of these domains, these did not translate into statistically significant between-group differences. Complete within-group data are provided in Supplementary Table 13.

### Exclusion of longitudinal PKG data

Longitudinal PKG outcomes were planned but excluded from analysis. The PKG is validated for continuous 6-10-day wear windows^27^, whereas approximately half of participants had incomplete wear across the 13-week follow-up due to charging and non-wear periods, and the remainder did not share a common analysable window relative to week-13. Post hoc subset analysis across 10 PKG endpoints in 48 participants would carry a risk of Type I error inflation. Future trials should pre-specify minimum continuous-wear requirements and standardised recording windows^28^.

## Discussion

In this pilot, double-blind, multicentre RCT CUE1+ was usable, safe, and well tolerated, with exploratory signals of benefit in motor severity, gait, sleep, and QoL. Although the study was not powered to assess clinical efficacy, and these findings should be interpreted as exploratory signals requiring confirmation in larger, adequately powered trials; yet they represent an important step toward defining the role of wearable vibrotactile cueing as an adjunct to standard care in PD. This safety and tolerability profile is consistent with previous findings for CUE1/CUE1+ in PD^24^ and orthostatic tremor^25^.

Dopaminergic therapy and DBS remain the therapeutic cornerstones of PD management^6,7,10,12,29^. Yet significant unmet needs persist. DBS reliably reduces motor fluctuations and dyskinesias, but residual axial symptoms and non-motor burdens often remain problematic^10,12,29,30^ highlighting the need for complementary interventions. Our findings suggest that vibrotactile stimulation could address domains not consistently improved by levodopa or DBS, including overall motor severity and patient-perceived QoL, without introducing the risks of invasive procedures. Whether benefits extend to gait variability and axial features, domains less consistently improved by dopaminergic therapy and DBS, remains an important question for future adequately powered trials.

The magnitude of motor improvement observed in the active group was notable. However, given the pilot sample size and exploratory design, this may reflect a combination of treatment effect, placebo response, and sampling variability. The findings nonetheless exceeded what would typically be expected from placebo alone^31,32^. Exploratory analyses revealed a differential pattern of sub-score response. Bradykinesia-related items demonstrated greater and more consistent improvement than tremor and rigidity sub-scores, which showed smaller and more variable changes. This differential response is consistent with the distinct pathophysiological substrates of these motor signs. Bradykinesia reflects impaired movement initiation and amplitude scaling driven by disrupted basal ganglia output and pathological beta-band synchronisation in the cortico-subthalamic circuit; external rhythmic vibrotactile stimulation may partially compensate for this by providing somatosensory input that engages the supplementary motor area and cerebellar-thalamic networks to restore temporal motor sequencing^12,16,33^. Parkinson’s-related tremor, by contrast, arises partly through distinct cerebello-thalamo-cortical oscillatory circuits less directly modulated by external rhythmic sensory entrainment, while rigidity, primarily mediated by altered muscle tone and supraspinal stretch reflex changes, may similarly be less amenable to cue-based intervention^2,12,34^. These data-driven mechanistic inferences are necessarily exploratory, and dedicated sub-score analyses in future adequately powered trials are needed to confirm this pattern.

The above observations align with neurophysiological work supporting cortical compensation and beta-band desynchronisation as plausible mediators of tactile cueing, mechanisms that conceptually align vibrotactile devices with other neuromodulatory strategies such as adaptive DBS^16,29,30,33,35–38^. A 2025 narrative review of 49 peer-reviewed studies encompassing 1,352 people with PD reported significant motor improvements in 78% of studies across vibrotactile paradigms, providing independent contextual support for this intervention modality^39^.

A strength of this study was the favourable compliance and participant experience, contrasting with the attrition rates often reported in exercise and pharmacological interventions for PD, where dropout may exceed 20%^36,40^. This may support the feasibility of discreet, home-based devices in real-world care. Nevertheless, the divergence between substantial clinician-rated improvements and more modest patient-reported impressions is consistent with reports of impaired self-awareness of motor symptoms (motor-agnosia) in PD^41^ and influence of patient expectations on perceived benefit^42^, highlighting the need for future endpoints that reflect outcomes that matter to patients and caregivers.

Although 43.5% of participants using the sham device met the MCID threshold on the MDS-UPDRS Part III, consistent with known placebo responses in PD when exposed to interventions^14,36–38^, a greater proportion achieving this in the active device group (84%) supports a genuine therapeutic effect. The MCID debate is ongoing, with thresholds ranging from −3.25 to −5.0 depending on methodology and baseline severity^43,44^, pointing out the need for anchor-based and patient-centred approaches in future studies.

Several limitations warrant consideration. The small sample and 13-week duration preclude definitive conclusions, consistent with the exploratory role of pilot RCTs^36,45^. Assessments were performed ON-medication to reflect daily function, though this may reduce between-group differences and limit comparability with DBS trials emphasising OFF-state testing^7,10,30^. ON-state was chosen since the device supplements dopaminergic therapy and OFF visits impose burden, disability, and anxiety^46^. Formal time-of-day standardisation was not pre-specified. Future trials should control for both post-dose timing and time of day to reduce diurnal variability in motor assessments. Although device-delivered therapies (e.g., subcutaneous apomorphine infusion or levodopa-carbidopa intestinal gel) were not explicit exclusion criteria, their use is unlikely given the mild-to-moderate disease of the cohort, modest LEDD, and stable oral therapy. Future definitive trials should specify this in eligibility criteria and include both medication states^30^. The relatively high baseline MDS-UPDRS III scores despite a median disease duration of five years likely reflect cohort phenotype and ethnic diversity, as all participants were levodopa-responsive and recent East London data show greater motor severity in South Asian and Black patients compared with White patients at similar durations^47^. A more fundamental limitation is that our sham was non-vibrating, and therefore could not fully equate cutaneous sensation, expectancy, or blinding between arms^34,36^. Formal participant-level blinding was not assessed. Definitive trials should employ an active sham matched on perceived sensation with validated blinding-assessment questionnaires. The greater improvement in bradykinesia versus tremor and rigidity supports a mechanism-specific rather than expectation-driven effect, consistent with evidence that open-loop vibrotactile stimulation does not reduce tremor power^34^. Offering the CUE1+ device to all participants post-trial, though ethically appropriate and pre-specified in the protocol^48^, may have introduced recruitment or response bias, which future trials should address. Compliance and satisfaction were self-reported, which may bias results, though digital usage logs could mitigate this. Finally, stimulation parameters were fixed; adaptive or patient-tailored paradigms, analogous to adaptive DBS, may optimise outcomes^29,35^. Future trials should include assessments both with and without device use to distinguish acute symptomatic effects from sustained carry-over benefits and better define mechanism and duration of action^17,18^. Longer follow-up is also needed to assess durability, falls, hospital admissions, caregiver burden, and cost-effectiveness across diverse populations^5,24,36,40,49,50^. In conclusion, CUE1+ was feasible, safe, and well tolerated over 12 weeks. The exploratory signals on MDS-UPDRS Part III and PDQ-39 are encouraging but, given the pilot size and non-vibrating sham, do not establish clinical efficacy or therapeutic benefit. Adequately powered double-blind trials with an active vibrotactile sham and validated blinding assessment are required.

## Methods

### Study design and settings

This multi-centre, double-blind, pilot RCT involved a 12-week intervention with assessments at baseline (week 0) and week 13. This study was conducted in accordance with the Declaration of Helsinki. Ethical approval was granted by the London-Dulwich Research Ethics Committee (reference 23/PR/1526; approval date 22 February 2024), which covered all participating sites. This trial was registered prospectively with ClinicalTrials.gov (registration number: NCT06174948; date of registration: 13 February 2024). Written informed consent was obtained from all participants prior to any study-related procedures. The protocol and statistical analysis plan were finalised before enrolment and are published^48^. The CONSORT checklist is provided in Supplementary Table 1.

### Participants

Participants were identified from the Neurology Department at Barts Health NHS Trust and the Department of Care of the Elderly at Homerton Healthcare NHS Foundation Trust. All study assessments were conducted at Queen Mary University of London (QMUL). Written informed consent was obtained from all participants before any study-related procedures were undertaken.

Eligible participants met the following inclusion criteria: a clinical diagnosis of PD according to the Movement Disorder Society criteria (MDS)^4^, age≥18 years, capacity and willingness to participate, and provision of written informed consent following review of the participant information sheet. Exclusion criteria included the presence of any medical or neurological conditions other than PD that could interfere with movement, balance, or independent participation in the trial. These included atypical parkinsonian syndromes, osteoarticular disorders, unstable antiparkinsonian medication within the prior 3 months, significant visual problems, audio-vestibular impairments, and a clinical diagnosis of dementia, including Alzheimer’s disease. Participants were also excluded if they were receiving any non-standard therapeutic, cueing, or vibrotactile stimulation interventions. Additional exclusion criteria comprised the presence of a) implanted metallic or electronic devices such as DBS; b) known hypersensitivity to vibrotactile stimulation; and/or c) dermatological conditions or open wounds at or near the intended site of device application (i.e., the sternum).

Demographics, disease duration, Hoehn and Yahr staging^26^, and baseline levodopa equivalent daily dose (LEDD) were collected. The Montreal Cognitive Assessment (MoCA)^51^ was administered at baseline to characterise participants’ cognitive profiles and support secondary analyses adjusting for cognitive status. Participants were excluded only if they had a prior diagnosis of dementia or Alzheimer’s disease; a MoCA score <26/30 was not exclusionary, reflecting the prevalence of mild cognitive impairment in Parkinson’s disease and the aim of an inclusive, generalisable trial design. Both the use of the MoCA and this threshold were pre-specified in the published trial protocol^48^. Sex and race/ethnicity were self-reported using UK Office for National Statistics categories^52^, recorded for descriptive purposes only, and reported in alphabetical order with ‘other’ components detailed in the table footnote.

### Interventions

Participants were randomised (1:1) to active or sham device. The active CUE1+ delivers vibrotactile stimulation at 80% maximum intensity with 800-ms pulse/rest intervals. These settings (vibration frequency 145 Hz ±10%, amplitude modulated at 0.625 Hz with an 800 ms pulse and 800 ms rest cycle, at 80% intensity) were based on our prior feasibility study, which showed tolerability and promising motor benefit^24^, and align with the manufacturer’s default configuration developed through user testing in people with PD and pre-specified in the published trial protocol^48^. The sham device was identical in appearance but delivered no vibration. A detailed photograph and schematic description of the CUE1+ device is included (Supplementary Fig. 1), along with educational demonstration videos showing device setup and use in people with PD (Supplementary Table 2). Devices were worn on the sternum with dermatology-approved adhesive patches for 8 h daily over 12 weeks, reflecting the typical waking activity period in PD and consistent with our preceding feasibility study^24^, the maximum duration feasible within the project timeline, pre-specified in the published protocol^48^, and selected to provide a meaningful window for detecting sustained changes in motor and patient-reported outcomes in this chronic condition.

To preserve blinding, participants were told that stimulation might be subtle or become less perceptible with habituation. Both arms received identical scripted explanations. At the week-13 assessment, participants were instructed to remove the device ≥ 2 h before testing to minimise tactile after-effects and protect masking. This served two purposes: to protect assessor blinding, as an active device would reveal allocation, and to evaluate whether 12 weeks of daily use produced sustained functional improvement beyond acute stimulation, a more rigorous and clinically meaningful endpoint consistent with standard evaluation of neuroplastic and neuromodulatory interventions^17,18^.

Parkinson’s medications were required to be stable for ≥3 months prior to enrolment. Any changes prompted withdrawal from the randomised phase at which point data collection ceased. Consent was obtained to retain data collected up to withdrawal. Participants could also withdraw at any time, with previously collected data retained if they agreed. All protocol deviations and violations were reported to the study sponsor. Following unblinding, Group A participants were informed of their allocation, asked to return the sham device, and offered an active CUE1+ for use outside the trial. All participants were offered the CUE1+ upon trial completion, irrespective of group.

### Safety monitoring and device function

Adverse events (AEs) were captured weekly via participant diaries and at study visits, and were graded by severity (mild, moderate, severe), seriousness, and relatedness (unrelated, possible, probably related). Device malfunctions were pre-defined as failure to deliver programmed vibration pattern or amplitude, charging failure, or hardware fault. Active arm participants performed a daily ‘device function check’ to check device function. Sham-arm participants performed a daily ‘lightning test’ to check device function. Sham-arm participants were told that upon switching on their fully charged device if the light was blue, the device was working normally. Suspected malfunctions in either arm were verified by two team members, an unblinded technician and an unblinded researcher and promptly replaced. To protect assessor blinding, the blinded rater was not involved in managing participant-reported concerns, side effects, or device-related queries at any point during the trial. All such contacts were directed exclusively to the unblinded researcher and unblinded technician.

### Outcomes

This trial involved a 12-week intervention with assessments at baseline (week 0) and week 13. Primary outcomes were usability (recruitment, compliance, retention).

Participant-level usability included compliance to the allocated intervention, retention through the 13-week follow-up, and completeness of baseline and outcome data. Based on previous studies^24,25^, pre-specified success criteria were recruitment of ≥70% of eligible participants, retention of ≥75% completing the week-13 assessment, and compliance of ≥70% achieving ≥8 h daily use (Likert score 4). Safety and tolerability were defined by the absence of serious device-related adverse events, with all events prospectively captured, graded by severity and relatedness, and reported as described above. Compliance with the assigned 12-week intervention was assessed using two self-report measures. Participants were first asked to indicate their usage frequency of the CUE1+ device on a 5-point Likert scale ranging from 0 (did not use it at all) to 4 (used it daily as advised, i.e., 8+ h/day). Additionally, they were asked to provide a subjective rating of their overall compliance on a continuous scale from 0 (not compliant at all) to 10 (completely compliant). Safety and tolerability were monitored throughout, including any clinical events and adverse events as described above. In the end of the study, all participants completed a satisfaction questionnaire on their experience with CUE1 + , as published with the trial protocol^48^. The questionnaire utilised a 5-point Likert scale, ranging from 0 (not at all) to 4 (extremely), to assess various aspects of participant satisfaction. Scores for all endpoints were subsequently compared between groups.

Clinical visits were conducted at baseline (week 0) and follow-up (week 13), each lasting approximately half a day. These visits included motor assessment and completion of self-reported outcome questionnaires. To mitigate fatigue-related bias, the order of assessments was randomised, and rest breaks were offered. The full assessment schedule is available in the published protocol^48^.

Secondary exploratory outcomes included exploratory clinical outcome measures intended to inform the design of future definitive trials rather than to establish clinical efficacy. Details on all clinical efficacy measures, scoring procedures, minimal clinically important differences (MCIDs) and additional reference list are provided (Supplementary Table 3). These outcomes were evaluated to inform the design of a future definitive RCT. Motor assessments included the gold-standard clinician-rated motor examination in PD, the Movement Disorder Society-sponsored revision of the Unified Parkinson’s Disease Rating Scale (MDS-UPDRS) Part III^53^, which comprises 33 clinician-rated items (0-4 each; total 0-132), and was assessed by trained raters masked to allocation, and Functional Gait Assessment^54^, Timed Up and Go (TUG)^55^, TUG with dual-task using serial sevens, and the two keyboard-based tapping tests to measure hand dexterity and movement (e.g., kinesia), Bradykinesia Akinesia INcoordination (BRAIN-Kinesia Score; KS)^56^ and Digital Finger Tapping (DFT-KS)^57^ tests for the most affected side. For MDS-UPDRS Part III, exploratory analyses summarised bradykinesia, rigidity, tremor and axial sub-scores, following conventional groupings. Optional video recordings of the MDS-UPDRS Part III assessments were retained for educational purposes to QMUL, with participant consent. All motor assessments were performed ON-medication (45-60 minutes post-levodopa dose) to reflect daily life and reduce OFF burden, consistent with our protocol^24,48^. Because ON-state testing can interact with levodopa responsiveness, baseline LEDD was recorded (Table 1) and Follow-up analyses adjusted for baseline MDS-UPDRS III. Parkinson’s KinetiGraph (PKG)^27^ wristwatches (Supplementary Table 3) were worn continuously from baseline to week 13.

Patient-reported outcomes covered both motor and non-motor domains and included the MDS-UPDRS Parts I, II, and IV^53^, Activities-specific Balance Confidence Scale (ABC)^58^, Pittsburgh Sleep Quality Index (PSQI)^45^, Parkinson’s Disease Questionnaire-39 (PDQ-39)^59^, Hospital Anxiety and Depression Scale (HADS)^43^, Fatigue Severity Scale (FSS)^44^, and Patient Global Impression of Change^40^. For PGIC, the prespecified item was ‘Change Since Beginning of Care’ (1=much worse to 7=much better). The ‘Degree of Change’ item (1=unchanged to 5=very much changed) was exploratory. Higher scores indicate greater improvement for the former and greater perceived change (not necessarily improvement) for the latter.

### Randomisation and masking

At the time of protocol publication^48^, treatment allocation was masked. After trial completion, Group A was unmasked as the sham intervention and Group B as the active non-invasive vibrotactile device. Masking of participants, investigators and assessor was maintained throughout the study until completion of data collection and analysis. Randomisation was done centrally using a secure, web-based system (www.sealedenvelope.com), employing permuted blocks of varying sizes (2, 4, 6) to ensure allocation concealment and minimise predictability (Supplementary Table 4). Multiple pre-labelled randomisation sequences (1-4) and block identifiers (1-14) were used to track allocation without compromising blinding. One researcher (CS), not involved in assessments or analysis, managed the randomisation process and held exclusive access to allocation codes. These codes were securely stored and inaccessible to other team members. No emergency unblinding occurred or was anticipated, owing to the non-invasive nature of the intervention and the absence of adverse events in the feasibility study^24^.

### Statistical analysis

Statistical analyses were conducted using IBM SPSS Statistics (version 29; IBM Corp., Armonk, NY, USA). Descriptive statistics summarised baseline characteristics and outcomes. For continuous variables, normally distributed data are presented as mean (standard deviation; SD), and non-normally distributed data as median (interquartile range; IQR). Categorical variables are shown as absolute (n) and relative (%) values. Normality was assessed using the Shapiro-Wilk test, supported by histogram and Q-Q plot inspection. Ninety-five per cent confidence intervals (95%CI) are reported where appropriate to indicate precision.

Between-group comparisons at baseline and follow-up used independent-samples t tests for normally distributed variables and Mann-Whitney U tests for non-normally distributed variables. Within-group comparisons used paired t-tests (normal distribution) or Wilcoxon signed-rank tests (non-normal distribution). When one timepoint was normally distributed and the other was not, non-parametric methods were applied. An intention-to-treat (ITT) approach was used for all clinical efficacy outcomes. No data imputation was performed. Participants who discontinued contributed data up to withdrawal, and these were included where available. Missing follow-up data, including incomplete PKG recordings, were not imputed; analyses were restricted to observed cases. The absence of longitudinal PKG accelerometry data, which could have objectively supported clinician-rated motor findings, is a notable limitation. Future trials should pre-specify minimum wear requirements and standardised recording windows to ensure analysable data. A Bonferroni correction was pre-specified for the 14 secondary exploratory outcomes (Table 3), adjusting the significance threshold from *p* < 0.05 to *p* < 0.004 (0.05 ÷ 14). The MDS-UPDRS Part III, as the primary exploratory outcome, was assessed at *α* = 0.05 without correction, and results meeting the Bonferroni threshold are clearly indicated in the Results section.

### Sample size

As a pilot RCT, no a priori power calculation was performed. The sample size was guided by Sim and Julius^60^, who recommend 20–75 participants per arm to detect moderate to large effects. Drawing from feasibility data, including a previous single-arm study^24^ involving ten participants, the target sample was initially set at 20 per group. To account for an anticipated dropout rate of up to 25%, consistent with attrition rates reported in comparable device trials^36–38^ and reflecting the general burden of any long-term trial participation rather than expectation of allocation detection, the final recruitment goal was increased to 25 participants per group. Analysis of covariance (ANCOVA) was performed with follow-up MDS-UPDRS III as the dependent variable, group as the fixed factor, baseline MDS-UPDRS III scores, age, and LEDD as covariates, with robust standard errors. This ANCOVA was pre-specified for MDS-UPDRS Part III as the primary exploratory outcome and is reported alongside the unadjusted between-group comparison as a sensitivity analysis. A supportive ANCOVA was additionally performed for PDQ-39 SI, the only other outcome meeting the Bonferroni-corrected threshold, using an equivalent covariate structure (baseline PDQ-39 SI, age, LEDD).

### Ethics approval and consent to participate

The study was approved by the London-Dulwich Research Ethics Committee (reference 23/PR/1526; approval date 22 February 2024), which covered all participating sites. All participants provided written informed consent prior to enrolment.

## Supplementary information

Supplementary File

## Data Availability

Data availabilityThe de-identified individual participant dataset generated and analysed during the current study, together with the data dictionary, study protocol, statistical analysis plan, and informed consent form, are not publicly available due to privacy restrictions under the UK Data Protection Act 2018 and GDPR, but are available from the corresponding author (C.S., c.simonet@qmul.ac.uk) on reasonable request and following completion of a data use agreement, in line with ICMJE data sharing guidelines.

## Code availability

No custom computer code or scripts were generated for this study. All statistical analyses were performed using IBM SPSS Statistics version 29 (IBM Corp., Armonk, NY, USA) using standard built-in procedures. Analysis syntax files are available from the corresponding author on reasonable request.
