# Supplementary material for: Sternum-worn vibrotactile device in Parkinson’s disease: a randomised, double-blind, placebo-controlled pilot trial
Source: NPJ Parkinsons Dis. 2026 Jul 3;12:232. doi: 10.1038/s41531-026-01448-y (PMC13612825; doi:10.1038/s41531-026-01448-y)
Supplement: Supplementary file 1 [file 41531_2026_1448_MOESM1_ESM.pdf]

Supplementary File

Contents

Supplementary File 1

Supplementary Table 1. CONSORT 2025 Checklist of the study 2

Supplementary Figure 1. The CUE1+ device 5

Supplementary Table 2. Supplementary Multimedia Resources (Educational Videos) 6

Supplementary Table 3. Description of motor and non-motor outcomes 7

Supplementary Table 4. Randomisation list 15

Supplementary Table 5. Participant-reported experience with the CUE1+ intervention 17

Supplementary Table 6. Between-group baseline scores of clinical effectiveness outcomes ... 18

Supplementary Table 7. Between-group baseline scores for Parkinson's KinetiGraph outcomes 19

Supplementary Table 8. ANCOVA of MDS-UPDRS Part III scores at follow-up adjusted for baseline MDS-UPDRS Part III, age, and LEDD 20

Supplementary Table 9. ANCOVA of PDQ-39 Summary Index at follow-up adjusted for baseline PDQ-39 SI, age, and LEDD 21

Supplementary Table 10. Between-group baseline scores for MDS-UPDRS Part III subscores (items 3.1-3.18) 22

Supplementary Table 11. Between-group follow-up scores for MDS-UPDRS Part III subscores (items 3.1-3.18) 24

Supplementary Table 12. Within-group change in MDS-UPDRS Part III subscores (items 3.1-3.18) 26

Supplementary Table 13. Within group difference in outcomes at follow up 28

Supplementary Table 1. CONSORT 2025 Checklist of the study

Section/topic	No	CONSORT 2025 checklist item description	Reported on page no.
**Title and abstract**			
Title and structured abstract	1a	Identification as a randomised trial	1-2
	1b	Structured summary of the trial design, methods, results, and conclusions	2
**Open science**			2
Trial registration	2	Name of trial registry, identifying number (with URL) and date of registration	
Protocol and statistical analysis plan	3	Where the trial protocol and statistical analysis plan can be accessed	17 (protocol published; reference number 26 in manuscript)
Data sharing	4	Where and how the individual de-identified participant data (including data dictionary), statistical code and any other materials can be accessed	20
Funding and conflicts of interest	5a	Sources of funding and other support (eg, supply of drugs), and role of funders in the design, conduct, analysis and reporting of the trial	20
	5b	Financial and other conflicts of interest of the manuscript authors	20
**Introduction**			3-4
Background and rationale	6	Scientific background and rationale	
Objectives	7	Specific objectives related to benefits and harms	4
**Methods**			
Patient and public involvement	8	Details of patient or public involvement in the design, conduct and reporting of the trial	11
Trial design	9	Description of trial design including type of trial (eg, parallel group, crossover), allocation ratio, and framework (eg, superiority, equivalence, non-inferiority, exploratory)	11-19
Changes to trial protocol	10	Important changes to the trial after it commenced including any outcomes or analyses that were not prespecified, with reason	7
Trial setting	11	Settings (eg, community, hospital) and locations (eg, countries, sites) where the trial was conducted	11-13
Eligibility criteria	12a	Eligibility criteria for participants	11-13
	12b	If applicable, eligibility criteria for sites and for individuals delivering the interventions (eg, surgeons, physiotherapists)	n/a
Intervention and comparator	13	Intervention and comparator with sufficient details to allow replication. If relevant, where additional materials describing the intervention and comparator (eg, intervention manual) can be accessed	13-14
Outcomes	14	Prespecified primary and secondary outcomes, including the specific measurement variable (eg, systolic blood pressure), analysis metric (eg, change from baseline, final value, time to event), method of aggregation (eg, median, proportion), and time point for each outcome	15-17
Harms	15	How harms were defined and assessed (eg, systematically, non-systematically)	15-17
Sample size	16a	How sample size was determined, including all assumptions supporting the sample size calculation	19
	16b	Explanation of any interim analyses and stopping guidelines	n/a
Randomisation:			17-18
	17a	Who generated the random allocation sequence and the method used	

Sequence generation	17b	Type of randomisation and details of any restriction (eg, stratification, blocking and block size)	17-18
			**Reported on page no.**
Allocation concealment mechanism	18	Mechanism used to implement the random allocation sequence (eg, central computer/telephone; sequentially numbered, opaque, sealed containers), describing any steps to conceal the sequence until interventions were assigned	17-18
Implementation	19	Whether the personnel who enrolled and those who assigned participants to the interventions had access to the random allocation sequence	17-18
Blinding	20a	Who was blinded after assignment to interventions (eg, participants, care providers, outcome assessors, data analysts)	17-18
	20b	If blinded, how blinding was achieved and description of the similarity of interventions	17-18
Statistical methods	21a	Statistical methods used to compare groups for primary and secondary outcomes, including harms	18-19
	21b	Definition of who is included in each analysis (eg, all randomised participants), and in which group	18-19
	21c	How missing data were handled in the analysis	18-19
	21d	Methods for any additional analyses (eg, subgroup and sensitivity analyses), distinguishing prespecified from post hoc	18-19
**Results**			
Participant flow, including flow diagram	22a	For each group, the numbers of participants who were randomly assigned, received intended intervention, and were analysed for the primary outcome	4
	22b	For each group, losses and exclusions after randomisation, together with reasons	4
Recruitment	23a	Dates defining the periods of recruitment and follow-up for outcomes of benefits and harms	4
	23b	If relevant, why the trial ended or was stopped	n/a
Intervention and comparator delivery	24a	Intervention and comparator as they were actually administered (eg, where appropriate, who delivered the intervention/comparator, how participants adhered, whether they were delivered as intended (fidelity))	5-7
	24b	Concomitant care received during the trial for each group	n/a
Baseline data	25	A table showing baseline demographic and clinical characteristics for each group	28
Numbers analysed, outcomes and estimation	26	For each primary and secondary outcome, by group:<br>● the number of participants included in the analysis<br>● the number of participants with available data at the outcome time point<br>● result for each group, and the estimated effect size and its precision (such as 95% confidence interval)<br>● for binary outcomes, presentation of both absolute and relative effect size	28-32 and Supplementary Tables 5-13
Harms	27	All harms or unintended events in each group	4
Ancillary analyses	28	Any other analyses performed, including subgroup and sensitivity analyses, distinguishing pre-specified from post hoc	6; Supplementary tables 8-9
**Discussion**			7-11
Interpretation	29	Interpretation consistent with results, balancing benefits and harms, and considering other relevant evidence	7-11
Limitations	30	Trial limitations, addressing sources of potential bias, imprecision, generalisability, and, if relevant, multiplicity of analyses	10-11

Citation: Hopewell S, Chan AW, Collins GS, Hróbjartsson A, Moher D, Schulz KF, et al. CONSORT 2025 Statement: updated guideline for reporting randomised trials. BMJ. 2025; 388:e081123. https://dx.doi.org/10.1136/bmj-2024-081123

© 2025 Hopewell et al. This is an Open Access article distributed under the terms of the Creative Commons

Attribution License (https://creativecommons.org/licenses/by/4.0/), which permits unrestricted use, distribution, and reproduction in any medium, provided the original work is properly cited.

*We strongly recommend reading this statement in conjunction with the CONSORT 2025 Explanation and Elaboration and/or the CONSORT 2025 Expanded Checklist for important clarifications on all the items. We also recommend reading relevant CONSORT extensions. See www.consort-spirit.org.

Supplementary Figure 1. The CUE1+ device

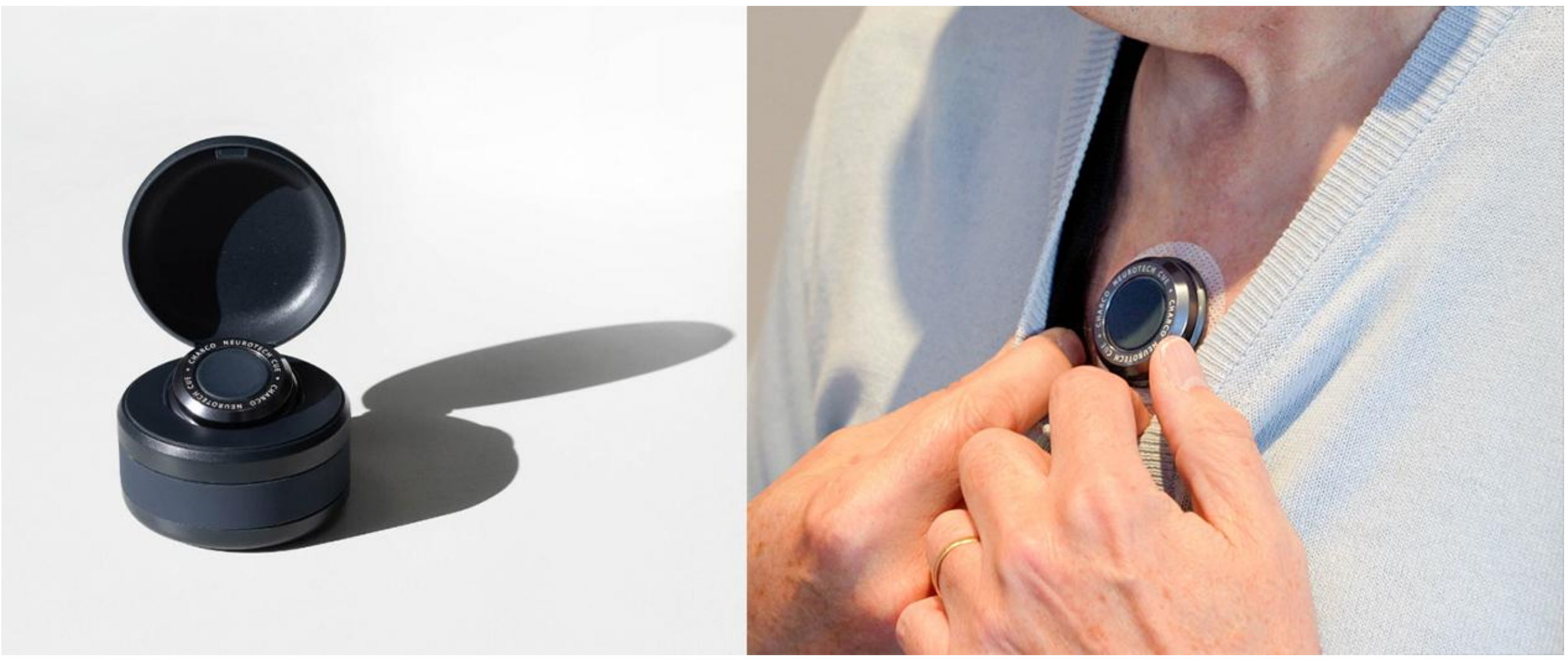

The CUE1+ is a non-invasive medical device designed to support the management of both motor and non-motor symptoms in people living with Parkinson’s. The device is compact and discreet (dimensions: 40 mm in diameter, 11 mm in height; weight: 17 g) and is affixed to the sternum using dermatologically tested adhesive patches. While it is water-resistant, it is not waterproof and should be removed prior to showering. When activated, the device delivers targeted vibrotactile stimulation via a silent motor, employing a proprietary waveform informed by extensive user feedback and clinical research. It integrates low-frequency auditory cueing with high-frequency vibrotactile stimulation, delivered in a structured pattern intended to optimise therapeutic benefit. A sham version of the CUE1+ is used as a control within the trial. It is externally identical to the active device, but its therapeutic functions have been fully disabled.

Supplementary Table 2. Supplementary Multimedia Resources (Educational Videos)

**Title / Description**	**Link**
Professor James Kilner on how CUE1 may improve symptoms in Parkinson's disease	https://www.youtube.com/watch?v=Tpl9xtCnEqI
Set-up and how to use the CUE1+ device	https://www.youtube.com/watch?v=itHAd6KkO60&t=20s
Dr Alister Mackett on CUE1 Device, a Vibrational Therapy to Potentially Treat Parkinson Disease Symptoms	https://www.youtube.com/watch?v=qAUAmGOlILU
The Science of the CUE1/CUE1+	https://www.youtube.com/watch?v=OqMeiK3OIgI
CUE1/CUE1+ 'freeze of gait' reduction test	https://www.youtube.com/watch?v=N-x_yb1ykkM
CUE1/CUE1+ 'Timed Tapping' test	https://www.youtube.com/watch?v=McvgNyOV5FQ

Note: The following resources are provided for educational purposes only. They illustrate the scientific rationale, set-up, and practical use of the CUE1+ device. Two demonstration videos are included: one showing the effect of CUE1+ in a patient with slowed gait and freezing of gait during the Timed Up and Go (TUG) test, and another showing timed tapping to illustrate its effect on bradykinesia. CUE1 and CUE1+ are identical in terms of therapeutic parameters; the only difference is the extended battery life of CUE1+ compared with the earlier CUE1 model. Treatment settings are unchanged. The links are publicly available (e.g., YouTube) and contain no patient-identifiable information. Permission to reference these materials has been obtained from Charco Neurotech Ltd., the original creators. These resources are included solely for explanatory and educational purposes to support editors' and reviewers' understanding of the device and its proposed mechanisms; they are not presented for promotional purposes.

Supplementary Table 3. Description of motor and non-motor outcomes

Outcome	Outcome subcategories	Description	Scoring	MCID	References
MDS-UPDRS	Part I	Evaluates non-motor experiences of daily living through 13 questions, divided into Part IA (investigator-assessed) and Part IB (patient self-administered but reviewable by the rater).	Each MDS-UPDRS item is rated on a scale from 0 (normal) to 4 (severe), reflecting the impact of PD symptoms, with higher scores indicating greater severity.<br><br>Part I: Non-Motor Experiences of Daily Living<br>Score Range: 0-52<br><br>Part II: Motor Experiences of Daily Living<br>Score Range: 0-52<br><br>Part III: Motor Examination<br>Score Range: 0-108<br><br>Part IV: Motor Complications<br>Score Range: 0-28	Part I: Improvement: A decrease > 2.64 points<br><br>Part II: Improvement: A decrease > 3.05 points<br><br>Part III: Improvement: A decrease > 3.25 points<br><br>Part IV: Improvement: A decrease > 0.90 points	Goetz et al. [1]<br>Horváth et al. [2]<br>Horváth et al. [3]<br><br>Makkos et al. [4]
	Part II	Addresses motor experiences of daily living with 13 self-administered questions, reviewable by the rater.			
	Part III	Involves a motor examination with 33 scores based on 18 questions, completed by the rater following specific instruction.			
	Part IV	Covers motor complications with 6 questions, integrating patient information and rater observations.			
FGA	Not applicable	A 10-item test evaluating complex gait tasks like walking with head turns, walking and turning and climbing stairs.	It scores between 0 and 3 per item, with a maximum of 30 points, where higher scores indicate better performance.	An increase ≥ 4.3 points	Wrisley et al. [5]<br>Petersen et al. [6]
TUG	Not applicable	Assesses mobility, balance, walking ability, and fall risks.	The mean time in people with Parkinson's	A decrease > 3.5 s	Podsiadho et al. [7]<br>Huang et al. [8]

			has been reported between 10.3-14.8 seconds.		
BRAIN tap test	KS	Assesses upper limb motor function and has been validated in patients with Parkinson's and controls. Participants use the index finger of a single hand to alternately strike the 'S' and ';' keys on a standard computer keyboard, as fast and accurately as possible. The test is repeated for the other hand.	The number of key taps in 30 seconds (s)-higher scores indicate better performance.	The scale is used in PD. However, MCID is has not been yet established. Until an MCID is established, clinicians and researchers should consider the KS as one component of a comprehensive evaluation of motor function in PD.	Giovannoni et al. [9] Noyce et al. [10] Hasan et al. [11]
DFT	KS	The DFT test consists of a 20-second single key tapping test. Participants are instructed to repeatedly tap the down arrow key with their left index finger, as fast as possible for 20 s, whilst simultaneously depressing the left arrow key with their left middle finger. The same task is then repeated for the right hand. These instructions stabilise the wrist and forearm, isolating movement to the index finger metacarpal joint, thereby giving a true measurement of distal finger movement. This movement is also similar to the MDS- UPDRS finger tapping task where patients are asked to tap their index finger and thumb repeatedly.	KS is a measure of speed (number of keystrokes typed in 20 seconds-higher scores indicate better performance.	The scale is used in PD. However, MCID is has not been yet established.	Akram et al. [12]
ABC	Not applicable	A 16-item questionnaire which assesses self-perceived balance confidence in daily activities.	Scores range from 0-100. Cut-off score of 69%. Are predictive of recurrent falls.	The scale is used in PD. However, MCID is has not been yet established.	Mak & Pang et al. [13]

			(sensitivity: 93%, specificity: 69%)		
PSQI	Not applicable	Includes seven component scores: subjective sleep quality, sleep latency, sleep duration, habitual sleep efficiency, sleep disturbance, use of sleeping medication, and daytime dysfunction.	Scores range from 0-21 with a higher total score indicating worse sleep quality. In distinguishing good and poor sleepers, a global PSQI score > 5 yields sensitivity of 89.6% and specificity of 86.5%.	The scale is used in PD. However, MCID is has not been yet established. However, in Alzheimer's disease and dementia it has been reported as a decrease of ≥ 4.25 points.	Buysse et al. [14]<br>Teruel-Hernández E, et al. [15]
PDQ-39	SI<br>Mobility<br>ADL<br>Emotional Wellbeing<br>Stigma<br>Social Support<br>Cognition<br>Communication<br>Bodily Discomfort	A 39-item self-report questionnaire, which assesses how often people with Parkinson's experience difficulties across the 8 quality of life dimensions and impact of PD on specific dimensions of functioning and well-being.	PDQ-39 SI is calculated as an average of the scores from the eight domains of the questionnaire, each of which reflects a different aspect of health-related quality of life in PD. Dimension score is the sum of scores of each item in the dimension divided by the maximum possible score of all the items in the dimension, multiplied by 100. Lower scores reflect better quality of life.	PDQ-39 SI: Improvement: A decrease > 4.7 points<br><br>The domain-specific thresholds range as follows:<br>Mobility: -3.2 points<br>ADL: -4.4 points<br>Emotional Well-being: 4.2 points<br>Stigma: -5.6 points<br>Social Support: -11.4 points<br>Cognition: -1.8 points<br>Communication: -4.2 points<br>Bodily Discomfort: -2.1 points	Jenkinson et al. [16]<br>Horváth, K., et al. [17]
HADS	Anxiety	The HADS consists of 14 items, divided into two 7 item subscales. Anxiety items reflect a state of generalised anxiety.	The respondent rates each item on a 4-point scale ranging from 0 (absence) to	The scale is used in PD. However, MCID is has not been yet established.	Roberts et al. [18]

			3 (extreme presence). Scores of 0-7 are considered normal, 8-10 indicate borderline (mild) cases, and 11-21 suggest clinically significant (moderate to severe) anxiety or depression. A score of 8 or above on either subscale is commonly used as a cut-off for identifying possible cases.		
	Depression	Depression focuses on the concept of anhedonia	The respondent rates each item on a 4-point scale ranging from 0 (absence) to 3 (extreme presence). Similarly, as reposted above for anxiety component.	The scale is used in PD. However, MCID is has not been yet established.	Roberts et al. [18]
FSS	Not applicable	It is a 9-item scale which measures the severity of fatigue and its effect on a person's activities and lifestyle in patients with a variety of neurological disorders, including those with Parkinson's diagnosis.	The items are scored on a 7-point scale with 1 = strongly disagree and 7= strongly agree. The minimum score = 9 and maximum score possible = 63. Higher the score = greater fatigue severity.	The scale is used in PD. However, MCID is has not been yet established.	Hagell et al. [19]

PGIC	Change since care	The patient is asked to reflect on their experience since beginning treatment at this clinic. Specifically, they are prompted to describe any changes, positive or negative, in their activity limitations, symptoms, emotional wellbeing, and overall quality of life related to their painful condition.	Patients rate their overall change since starting care on a 1-7 scale, where 1 means no change or worse, and 7 indicates a very significant improvement.	The scale is used in PD. However, MCID is has not been yet established.	Hurst et al. [20]
	Degree of Change	The patient is asked to circle the number that best reflects their perceived degree of change in their condition since beginning care at this clinic. This self-assessment captures the overall impact of treatment from the patient's perspective.	Patients are asked to circle a number from 0 to 10 to reflect their overall change since starting care, where 0 indicates much better and 10 indicates much worse.	The scale is used in PD. However, MCID is has not been yet established.	Hurst et al. [20]
PKG-S	DK-50	The median dyskinesia score (DKS) for a subject during the recording period between 9am and 6pm. The DK_50 score measures the severity of dyskinesia at the 50th percentile (median).	The higher the DK_50 score the more severe the dyskinesia symptom is on average for this subject. The target score for DK_50 is ≤ 9.0.	The variable is used in PD but MICD has not been established.	Griffiths et al. [21] Kotschet et al. [22] Horne et al. [23] Braybrook et al. [24] Horne et al. [25]
	BK-50	The median bradykinesia score (BKS) for a subject during the recording period between 9am and 6pm. The BK_50 score measures the severity of bradykinesia at the 50th percentile (median).	The higher the BK_50 score the more severe the bradykinesia symptom is on average for this subject. The target score for BK_50 is ≤ 25.0.	The variable is used in PD but MICD has not been established.	Griffiths et al. [21] Kotschet et al. [22] Horne et al. [23] Braybrook et al. [24] Horne et al. [25]
	FDS	The Fluctuation Dyskinesia Score (FDS) for a subject during the recording period between 9am and 6pm. Quantifies the interquartile range (IQR) and the degree of	<7.5 is a non-fluctuator<br><br>7.5 to 13.0 is a controlled fluctuator (this is the	The variable is used in PD but MICD has not been established.	Griffiths et al. [21] Kotschet et al. [22] Horne et al. [23]

		symptom variability. Can be used to identify subjects with fluctuating symptoms.	target score range)<br><br>>13.0 is an uncontrolled fluctuator		Braybrook et al. [24]<br>Horne et al. [25]
	PCT-TM-TREMOR	The percentage of time tremor was detected for this subject during the recording period between 9am and 6pm. Indicates the absence or presence of tremor only. The unit of PCT_TM_TREMOR is percentage (%).	0 - 0.5% = No tremor<br><br>0.6 - 1% = Inconclusive<br><br>>1% = Tremor present	The variable is used in PD but MICD has not been established.	Griffiths et al. [21]<br>Kotschet et al. [22]<br>Horne et al. [23]<br>Braybrook et al. [24]<br>Horne et al. [25]
	PCT-TM-IMMOB	The percentage of time the subject was immobile during the recording period between 9am and 6pm (% of 2-minute awake time epochs with BKS >80) and is a measure of daytime somnolence. The unit of PCT_TM_IMMOB is percentage (%).	Higher values represent an increased percentage of time spent immobile. Percent time immobile	The variable is used in PD but MICD has not been established.	Griffiths et al. [21]<br>Kotschet et al. [22]<br>Horne et al. [23]<br>Braybrook et al. [24]<br>Horne et al. [25]
PKG-WS	Average Daily Step Count	Steps per day, averaged over the 7 days in the PKG session during 6am to 11pm each day.	Higher values are better.	The variable is used in PD but MICD has not been established.	Shokouhi et al. [26]
	Average Daily Long Walk	Average duration of periods of continuous walking, where each period is greater than 1 minute duration during 6am to 11pm each day. Lower average duration in long walks may indicate worsening disease symptoms or progressing gait issues. Calculated in seconds.	Higher values are better.	The variable is used in PD but MICD has not been established.	Shokouhi et al. [26]
PKG-RS	NIGHT-PCT-TM-SLEEP	Percentage of time the subject was asleep (BKS ≥80 in the majority of 14-minute intervals) during the recording period between 11pm and 6am. Higher values represent more time that the subject was asleep. The unit of NIGHT_PCT_TM_SLEEP is percentage (%).	The defined medians for control subjects and PD subjects are 64 and 54, respectively.	The variable is used in PD but MICD has not been established.	McGregor et al. [27]<br>Klingelhoefer et al. [28]
	NIGHT-SLEEP-QLTY	A score indicating the quality of the subject's sleep during the recording period between	Higher values will represent a percentage of time spent	The variable is used in PD but MICD has not	McGregor et al. [27]<br>Klingelhoefer et al. [28]

		11pm and 6am. The percentage is calculated as the time spent very immobile (BKS ≥ 110) relative to the time of immobility (BKS ≥ 80).	in higher stages of sleep (e.g., N3 or REM) during period of immobility (sleep).	been established.	
	TOT-SLEEP-SCORE	A score indicating the overall quality of subject's sleep during the recording period between 11pm and 6am. A higher value represents higher quality sleep on a scale of zero to twenty-seven (0-27). Made up of a combination of % Time Immobile, % Time Sleep and Sleep Quality. Indication of sleep efficiency where patient is sleeping for sustained periods and in higher stages of sleep; best predicts PSG.	A score lower than 24 is trending toward abnormal sleep (e.g., fewer periods of REM sleep and PDSS2 scores > 18).	The variable is used in PD but MICD has not been established.	McGregor et al. [27] Klingelhoefer et al. [28]

MCID minimal clinically important difference; MDS-UPDRS Movement Disorder Society-sponsored revision of the Unified Parkinson's Disease Rating Scale; PD Parkinson's disease; FGA Functional Gait Assessment; TUG Timed Up and Go; BRAIN Bradykinesia Akinesia Incoordination test; KS Kinesia Score; DFT Digital Finger Tapping; ABC Activities-specific Balance Confidence scale; PSQI Pittsburgh Sleep Quality Index; PDQ-39 Parkinson's Disease Questionnaire-39; SI summary index; ADL activities of daily living; HADS Hospital Anxiety and Depression Scale; FSS Fatigue Severity Scale; PGIC Patient Global Impression of Change; PKG Parkinson's KinetiGraph; PKG-S standard Parkinson's KinetiGraph scores; PKG-WS Parkinson's KinetiGraph gait/walking score; PKG-RS Parkinson's KinetiGraph overnight sleep score; DK-50 dyskinesia score (median); BK-50 bradykinesia score (median); FDS functional dyskinesia score; PCT-TM-TREMOR percentage of time with tremor; PCT-TM-IMMOB percentage of time immobile.

Reference list:

1. Goetz CG, Tilley BC, Shaftman SR, Stebbins GT, Fahn S, Martinez-Martin P et al (2008) Movement Disorder Society-sponsored revision of the Unified Parkinson's Disease Rating Scale (MDS-UPDRS): scale presentation and clinimetric testing results. Mov Disord 23:2129-2170
2. Horváth K, Aschermann Z, Kovács M, Makkos A, Harmat M, Janszky J et al (2017) Minimal clinically important differences for the experiences of daily living parts of the Movement Disorder Society-sponsored Unified Parkinson's Disease Rating Scale. Mov Disord 32:789-793
3. Horváth K, Aschermann Z, Ács P, Deli G, Janszky J, Komoly S et al (2015) Minimal clinically important difference on the motor examination part of the MDS-UPDRS. Parkinsonism Relat Disord 21:1421-1426
4. Makkos A, Kovács M, Pintér D, Janszky J, Kovács N (2019) Minimal clinically important difference for the historic parts of the Unified Dyskinesia Rating Scale. Parkinsonism Relat Disord 58:79-82
5. Wrisley DM, Marchetti GF, Kuharsky DK, Whitney SL (2004) Reliability, internal consistency, and validity of data obtained with the Functional Gait Assessment. Phys Ther 84:906-918
6. Petersen C, Steffen T, Paly E, Dvorak L, Nelson R (2017) Reliability and minimal detectable change for sit-to-stand tests and the Functional Gait Assessment in individuals with Parkinson disease. J Geriatr Phys Ther 40:223-226
7. Podsiadlo D, Richardson S (1991) The timed Up & Go: a test of basic functional mobility for frail elderly persons. J Am Geriatr Soc 39:142-148
8. Huang SL, Hsieh CL, Wu RM, Tai CH, Lin CH, Lu WS (2011) Minimal detectable change of the timed Up & Go test and the Dynamic Gait Index in people with Parkinson disease. Phys Ther 91:114-121
9. Giovannoni G, van Schalkwyk J, Fritz VU, Lees AJ (1999) Bradykinesia akinesia incoordination test (BRAIN test): an objective computerised assessment of upper limb motor function. J Neurol Neurosurg Psychiatry 67:624-629

10. Noyce AJ, Nagy A, Acharya S, Bestwick JP, Dobbs AJ, Lees AJ et al (2014) Bradykinesia-akinesia incoordination test: validating an online keyboard test of upper limb function. PLoS One 9:e96260
11. Hasan H, Burrows M, Athauda DS, Roussakis AA, Warner TT et al (2019) The BRadykinesia Akinesia INcoordination (BRAIN) tap test: capturing the sequence effect. Mov Disord Clin Pract 6:462-469
12. Akram N, Li H, Ben-Joseph A, Budu C, Gallagher DA, Bestwick JP et al (2022) Developing and assessing a new web-based tapping test for measuring distal movement in Parkinson's disease. Sci Rep 12:386
13. Mak MK, Pang MY (2009) Balance confidence and functional mobility are independently associated with falls in people with Parkinson's disease. J Neurol 256:742-749
14. Buysse DJ, Reynolds CF, Monk TH, Berman SR, Kupfer DJ (1989) The Pittsburgh Sleep Quality Index: a new instrument for psychiatric practice and research. Psychiatry Res 28:193-213
15. Teruel-Hernández E, López-Pina JA, Souto-Camba S, Báez-Suárez A, Medina-Ramírez R, Gómez-Conesa A (2023) Improving sleep quality, daytime sleepiness, and cognitive function in patients with dementia by therapeutic exercise and NESA neuromodulation: a multicentre clinical trial. Int J Environ Res Public Health 20:7027
16. Jenkinson C, Fitzpatrick R, Peto V, Greenhall R, Hyman N (1997) The Parkinson's Disease Questionnaire (PDQ-39): development and validation. Age Ageing 26:353-357
17. Horváth K, Aschermann Z, Kovács M, Makkos A, Harmat M, Janszky J et al (2017) Changes in quality of life in Parkinson's disease: how large must they be to be relevant? Neuroepidemiology 48:1-8
18. Roberts SB, Bonnici DM, Mackinnon AJ, Worcester MC (2001) Psychometric evaluation of the Hospital Anxiety and Depression Scale among female cardiac patients. Br J Health Psychol 6:373-383
19. Hagell P, Höglund A, Reimer J, Eriksson B, Knutsson I, Widner H, Cella D (2006) Measuring fatigue in Parkinson's disease: a psychometric study of two brief generic fatigue questionnaires. J Pain Symptom Manage 32:420-432
20. Hurst H, Bolton J (2004) Assessing the clinical significance of change scores recorded on subjective outcome measures. J Manipulative Physiol Ther 27:26-35
21. Griffiths RI, Kotschet K, Arfon S, Xu ZM, Johnson W, Drago J et al (2012) Automated assessment of bradykinesia and dyskinesia in Parkinson's disease. J Parkinsons Dis 2:47-55
22. Kotschet K, Johnson W, McGregor S, Hall C, Gschwandtner U, Horne M (2014) Daytime sleep in Parkinson's disease measured by episodes of immobility. Parkinsonism Relat Disord 20:578-583
23. Horne MK, McGregor S, Bergquist F (2015) An objective fluctuation score for Parkinson's disease. PLoS One 10:e0124522
24. Braybrook M, O'Connor S, Churchward P, Perera T, Farzanehfar P, Horne M (2016) An ambulatory tremor score for Parkinson's disease. J Parkinsons Dis 6:723-731
25. Horne M, Kotschet K, McGregor S (2016) The clinical validation of objective measurement of movement in Parkinson's disease. CNS 2:16-23
26. Shokouhi N, Khodakarami H, Fernando C, Osborn S, Horne M (2022) Accuracy of step count estimations in Parkinson's disease can be predicted using ambulatory monitoring. Front Aging Neurosci 14:904895
27. McGregor S, Churchward P, Soja K, Baque E, Macfarlane MD, McInerney J et al (2018) The use of accelerometry as a tool to measure disturbed nocturnal sleep in Parkinson's disease. NPJ Parkinsons Dis 4:1
28. Klingelhoefer L, Rizos A, Sauerbier A, McGregor S, Martinez-Martin P, Reichmann H et al (2016) Night-time sleep in Parkinson's disease: the potential use of Parkinson's KinetiGraph. Eur J Neurol 23:1275-1288

Supplementary Table 4. Randomisation list

block identifier	block size	sequence within block	treatment
1	4	1	Group A
1	4	2	Group A
1	4	3	Group B
1	4	4	Group B
2	6	1	Group B
2	6	2	Group B
2	6	3	Group B
2	6	4	Group A
2	6	5	Group A
2	6	6	Group A
3	4	1	Group B
3	4	2	Group A
3	4	3	Group B
3	4	4	Group A
4	2	1	Group A
4	2	2	Group B
5	2	1	Group B
5	2	2	Group A
6	2	1	Group B
6	2	2	Group A
7	6	1	Group B
7	6	2	Group B
7	6	3	Group A
7	6	4	Group B
7	6	5	Group A
7	6	6	Group A
8	6	1	Group A
8	6	2	Group B
8	6	3	Group B
8	6	4	Group A
8	6	5	Group B
8	6	6	Group A
9	2	1	Group B
9	2	2	Group A
10	4	1	Group B
10	4	2	Group A
10	4	3	Group B
10	4	4	Group A
11	2	1	Group B
11	2	2	Group A
12	4	1	Group A
12	4	2	Group A
12	4	3	Group B

12	4	4	Group B
13	2	1	Group B
13	2	2	Group A
14	4	1	Group A
14	4	2	Group B
14	4	3	Group B
14	4	4	Group A

Supplementary Table 5. Participant-reported experience with the CUE1+ intervention

Measure	Sham device group (n = 23)	Active device group (n = 25)	Between-group difference (95% CI)	P value
Perceived helpfulness for symptoms	0.0 (0.0-1.0)	2.0 (1.0-2.3)	-1.1 (-1.7 to -0.5)	0.001
Satisfaction with device use	2.0 (2.0-3.0)	3.0 (2.0-3.0)	1.0 (-0.5 to 1.0)	0.10
Likelihood of continued use	2.0 (2.0-3.0)	3.0 (3.0-3.3)	1.0 (0.5 to 1.0)	0.004
Likelihood of recommending the device	3.0 (2.0-3.0)	3.0 (2.0-3.0)	0.0 (0.0 to 1.0)	0.34

Data are presented as median (interquartile range).
Responses were recorded using a 5-point Likert scale ranging from 0 (lowest rating) to 4 (highest rating).
CI confidence interval.

Supplementary Table 6. Between-group baseline scores of clinical effectiveness outcomes

Measure	Sham device group (n = 23)	Active device group (n = 25)	Between-group difference (95% CI)	P value
MDS-UPDRS Part I	14.0 (10.0-21.0)	22.0 (14.0-26.0)	8.0 (1.0 to 12.0)	0.08
MDS-UPDRS Part II	16.0 (9.0-21.5)	21.0 (11.0-25.0)	5.0 (-5.0 to 14.0)	0.196
MDS-UPDRS Part III	44.0 (10.4)	43.4 (15.2)	0.64 (-6.7 to 8.0)	0.87
MDS-UPDRS Part IV	8.7 (5.3)	10.0 (4.6)	-1.26 (-4.1 to 1.6)	0.39
Functional Gait Assessment	17.0 (14.5-18.5)	18.0 (14.0-20.0)	-1.0 (-4.0 to 1.0)	0.57
Timed Up and Go, single task	10.4 (8.4-13.1)	10.0 (8.2-12.1)	0.4 (-1.6 to 2.9)	0.98
Timed Up and Go, dual task	14.2 (11.1-20.1)	13.2 (10.2-19.1)	1.0 (-4.0 to 5.9)	0.94
BRAIN tap KS (most affected side)	34.5 (31.0-46.8)	40.0 (37.0-46.0)	-5.5 (-12.0 to 5.0)	0.41
Digital finger tapping KS (most affected side)	67.5 (54.0-73.0)	64.0 (54.0-80.0)	3.5 (-18.0 to 14.0)	0.88
Activities-specific Balance Confidence	53.8 (41.9-87.8)	55.0 (41.3-80.0)	-1.3 (-15.0 to 31.3)	0.71
Pittsburgh Sleep Quality Index	10.9 (5.4)	10.9 (5.0)	-0.1 (-3.0 to 2.9)	0.97
PDQ-39 Summary Index	25.9 (11.5-43.4)	35.7 (27.9-51.1)	-9.7 (-24.4 to 7.0)	0.11
PDQ-39 Mobility	40.0 (6.3-63.0)	40.0 (22.5-65.0)	0.0 (-37.5 to 30.0)	0.40
PDQ-39 Activities of daily living	33.3 (8.3-47.9)	37.5 (16.7-62.5)	-4.2 (-37.5 to 16.7)	0.35
PDQ-39 Emotional wellbeing	20.8 (8.3-37.5)	41.7 (25.0-75.0)	-20.0 (-41.7 to -4.2)	0.008
PDQ-39 Stigma	12.5 (6.3-40.6)	25.0 (6.3-56.3)	-12.5 (-37.5 to 12.5)	0.14
PDQ-39 Social support	0.0 (0.0-16.7)	0.0 (25.0-25.0)	-8.3 (-16.7 to 8.3)	0.45
PDQ-39 Cognition	25.0 (15.6-40.6)	37.5 (25.0-56.3)	-12.5 (-31.3 to 6.3)	0.14
PDQ-39 Communication	16.7 (0.0-41.7)	33.3 (16.7-50.0)	-16.7 (-33.3 to 0.0)	0.08
PDQ-39 Bodily discomfort	50.0 (25.0-58.3)	33.3 (16.7-66.7)	16.7 (-25.0 to 25.0)	0.71
HADS Anxiety	6.0 (3.5-9.5)	11.0 (7.0-13.0)	-3.0 (-12.0 to 7.7)	0.005
HADS Depression	6.0 (4.0-10.0)	8.0 (6.0-10.0)	0.0 (-1.0 to 0.0)	0.25
Fatigue Severity Scale	36.0 (22.0-46.5)	38.0 (30.0-49.0)	-2.0 (-17.0 to 10.0)	0.52

Data are presented as median (IQR) or mean (SD), as appropriate. N denotes number; CI, confidence interval. MDS-UPDRS, Movement Disorder Society-Unified Parkinson's Disease Rating Scale; FGA, Functional Gait Assessment; TUG, Timed Up and Go; BRAIN tap, Bradykinesia Akinesia Incoordination tap test; KS, Kinesia Score; DFT, Digital Finger Tapping; ABC, Activities-specific Balance Confidence; PSQI, Pittsburgh Sleep Quality Index; PDQ-39, Parkinson's Disease Questionnaire-39; HADS, Hospital Anxiety and Depression Scale; FSS, Fatigue Severity Scale.

Supplementary Table 7. Between-group baseline scores for Parkinson's KinetiGraph outcomes

Measure	Sham device group (n = 23)	Active device group (n = 25)	Between-group difference (95% CI)	P value
PKG-S DK-50	1.4 (0.4-1.9)	1.4 (0.9-3.1)	0.0 (-54.4 to 9.4)	0.30
PKG-S BK-50	29.2 (24.6-36.4)	28.1 (26.6-32.6)	-1.1 (-19.0 to 20.9)	0.34
PKG-S FDS	8.1 (6.4-10.1)	8.0 (6.4-10.3)	-0.1 (-2.1 to 2.4)	0.86
PKG-S PCT-TM-TREMOR	2.7 (0.9-5.4)	1.8 (1.1-6.4)	-0.9 (-3.5 to 3.3)	0.96
PKG-S PCT-TM-IMMOB	9.0 (5.2-15.5)	10.5 (5.9-14.9)	1.6 (-6.7 to 7.0)	0.96
PKG-WS Average daily step count	4127.0 (2706.0-5828.8)	5528.0 (3807.0-8390.0)	1401.0 (-887.5 to 4128.0)	0.32
PKG-WS Average daily long walk	1057.0 (339.8-1676.3)	737.0 (416.0-2026.0)	-320.0 (-750.5 to 771.5)	0.94
PKG-RS NIGHT-PCT-TM-SLEEP	44.3 (35.5-56.4)	60.1 (46.8-73.4)	15.8 (1.9 to 32.6)	0.03
PKG-RS NIGHT-SLEEP-QLTY	59.0 (49.8-75.4)	73.6 (58.6-84.1)	14.7 (-0.8 to 27.5)	0.04
PKG-RS TOT-SLEEP-SCORE	1.5 (0.0-6.3)	7.0 (3.0-21.0)	5.5 (1.5 to 18.0)	0.03

Data are presented as median (IQR) or mean (SD), as appropriate. N denotes number; CI, confidence interval. PKG, Parkinson's KinetiGraph; PKG-S, standard PKG scores; PKG-WS, PKG walking score parameters; PKG-RS, overnight sleep scores; DK-50, dyskinesia score median; BK-50, bradykinesia score median; FDS, functional dyskinesia score; PCT-TM-TREMOR, percentage of time with tremor; PCT-TM-IMMOB, percentage of time immobile. The PKG wrist device was worn on the most affected side.

Supplementary Table 8. ANCOVA of MDS-UPDRS Part III scores at follow-up adjusted for baseline MDS-UPDRS Part III, age, and LEDD

Term	Estimate (β)	SE	95% CI	P value
Intercept	13.63	15.72	-17.19 to 44.44	0.386
Group (active vs sham)	-11.428	3.28	-17.85 to -4.98	<0.001
Baseline MDS-UPDRS Part III	0.66	0.15	0.36 to 0.96	<0.001
Baseline age	-0.05	0.20	-0.44 to 0.35	0.814
Baseline LEDD	0.00	0.01	-0.02 to 0.02	0.985

Data are presented as regression coefficients (β) with robust standard errors (SE) and 95% confidence intervals (CI). ANCOVA was performed with follow-up MDS-UPDRS Part III as the dependent variable, group as the fixed factor, and baseline MDS-UPDRS Part III, age, and levodopa equivalent daily dose (LEDD) as covariates, with robust standard errors. The adjusted means at covariate grand means (baseline MDS-UPDRS Part III = 43.7; age = 67.6 years; LEDD = 431 mg/day) were 27.9 (active group) and 39.4 (sham group). β, regression coefficient; SE, standard error; CI, confidence interval; LEDD, levodopa equivalent daily dose; MDS-UPDRS III, Movement Disorder Society-Unified Parkinson's Disease Rating Scale motor examination. P values are two-sided.

Supplementary Table 9. ANCOVA of PDQ-39 Summary Index at follow-up adjusted for baseline PDQ-39 SI, age, and LEDD

Term	Estimate (β)	SE	95% CI	P value
Intercept	10.21	11.08	-11.64 to 32.06	0.36
Group (active vs sham)	-7.21	3.62	-14.53 to 0.10	0.053
Baseline PDQ-39 SI	0.62	0.09	0.44 to 0.80	<0.001
Baseline age	-0.08	0.06	-0.20 to 0.04	0.19
Baseline LEDD	0.00	0.00	-0.01 to 0.01	0.83

Data are presented as regression coefficients (β) with robust standard errors (SE) and 95% confidence intervals (CI). ANCOVA was performed with follow-up PDQ-39 Summary Index as the dependent variable, group as the fixed factor, and baseline PDQ-39 Summary Index, age, and levodopa equivalent daily dose (LEDD) as covariates.

Supplementary Table 10. Between-group baseline scores for MDS-UPDRS Part III subscores (items 3.1-3.18)

Item	Sham device group (n = 23)	Active device group (n = 25)	Between-group difference (95% CI)	P value
3.1 Speech	1.00 (1.00-1.00)	1.00 (1.00-2.00)	0.00 (0.00 to 0.00)	0.64
3.2 Facial expression	2.00 (1.00-2.00)	2.00 (1.00-2.00)	0.00 (-1.00 to 1.00)	0.93
3.3a Rigidity - neck	2.00 (2.00-3.00)	2.00 (2.00-3.00)	0.00 (-1.00 to 1.00)	0.68
3.3b Rigidity - right upper extremity	2.00 (1.50-2.50)	2.00 (2.00-2.00)	0.00 (0.00 to 0.00)	0.90
3.3c Rigidity - left upper extremity	2.00 (1.00-3.00)	2.00 (0.00-3.00)	0.00 (-2.00 to 1.00)	0.68
3.3d Rigidity - right lower extremity	1.00 (1.00-2.00)	1.00 (1.00-2.00)	0.00 (-1.00 to 1.00)	0.92
3.3e Rigidity - left lower extremity	2.00 (1.00-2.00)	1.00 (0.00-2.00)	1.00 (-1.00 to 2.00)	0.30
3.4a Finger tapping - right hand	2.00 (2.00-3.00)	2.00 (2.00-3.00)	0.00 (-1.00 to 1.00)	0.76
3.4b Finger tapping - left hand	2.00 (1.00-2.00)	2.00 (1.00-3.00)	0.00 (-1.00 to 1.00)	0.86
3.5a Hand movements - right hand	2.00 (1.00-3.00)	2.00 (1.00-3.00)	0.00 (-1.00 to 1.00)	0.81
3.5b Hand movements - left hand	2.00 (1.00-2.00)	2.00 (1.00-2.00)	0.00 (-1.00 to 1.00)	0.99
3.6a Pronation-supination - right hand	2.00 (1.00-2.00)	2.00 (1.00-2.00)	0.00 (-1.00 to 1.00)	0.93
3.6b Pronation-supination - left hand	2.00 (1.00-2.00)	2.00 (1.00-2.00)	0.00 (-1.00 to 1.00)	0.89
3.7a Toe tapping - right foot	2.00 (1.00-3.00)	2.00 (1.00-3.00)	0.00 (-1.00 to 1.00)	0.98
3.7b Toe tapping - left foot	2.00 (1.00-3.00)	2.00 (1.00-2.00)	0.00 (-1.00 to 1.00)	0.92
3.8a Leg agility - right leg	2.00 (1.00-2.00)	2.00 (1.00-3.00)	0.00 (-1.00 to 1.00)	0.83
3.8b Leg agility - left leg	1.00 (0.00-2.00)	1.00 (0.00-2.00)	0.00 (-1.00 to 1.00)	0.98
3.9 Arising from chair	2.00 (1.00-2.00)	2.00 (1.00-2.00)	0.00 (-1.00 to 1.00)	0.87
3.10 Gait	2.00 (1.00-2.00)	2.00 (1.00-3.00)	0.00 (-1.00 to 1.00)	0.79
3.11 Freezing of gait	0.00 (0.00-0.00)	0.00 (0.00-0.00)	0.00 (0.00 to 0.00)	1.00
3.12 Postural stability	1.00 (0.00-2.00)	1.00 (0.00-2.00)	0.00 (-1.00 to 1.00)	0.91
3.13 Posture	2.00 (1.00-2.00)	2.00 (1.00-2.00)	0.00 (-1.00 to 1.00)	0.93
3.14 Global spontaneity of movement	2.00 (1.00-2.00)	2.00 (1.00-3.00)	0.00 (-1.00 to 1.00)	0.80
3.15a Postural tremor - right hand	0.00 (0.00-1.00)	0.00 (0.00-1.00)	0.00 (-1.00 to 0.00)	0.85
3.15b Postural tremor - left hand	0.00 (0.00-1.00)	0.00 (0.00-1.00)	0.00 (-1.00 to 1.00)	0.90
3.16a Kinetic tremor - right hand	0.00 (0.00-1.00)	0.00 (0.00-1.00)	0.00 (-1.00 to 1.00)	0.95
3.16b Kinetic tremor - left hand	0.00 (0.00-1.00)	0.00 (0.00-1.00)	0.00 (-1.00 to 1.00)	0.89
3.17a Rest tremor amplitude - right upper extremity	0.00 (0.00-0.00)	0.00 (0.00-0.00)	0.00 (0.00 to 0.00)	1.00
3.17b Rest tremor amplitude - left upper extremity	0.00 (0.00-0.00)	0.00 (0.00-0.00)	0.00 (0.00 to 0.00)	1.00
3.17c Rest tremor amplitude - right lower extremity	0.00 (0.00-0.00)	0.00 (0.00-0.00)	0.00 (0.00 to 0.00)	1.00
3.17d Rest tremor amplitude - left lower extremity	0.00 (0.00-0.00)	0.00 (0.00-0.00)	0.00 (0.00 to 0.00)	1.00
3.17e Rest tremor amplitude - lip/jaw	0.00 (0.00-0.00)	0.00 (0.00-0.00)	0.00 (0.00 to 0.00)	1.00
3.18 Constancy of rest tremor	1.00 (0.00-1.00)	1.00 (0.00-1.00)	0.00 (-1.00 to 1.00)	0.87

Data are presented as median (IQR), as appropriate. N denotes number of participants; CI, confidence interval. MDS-UPDRS, Movement Disorder Society-Unified Parkinson's Disease Rating Scale.

Supplementary Table 11. Between-group follow-up scores for MDS-UPDRS Part III subscores (items 3.1-3.18)

Item	Sham device group (n = 23)	Active device group (n = 25)	Between-group difference (95% CI)	P value
3.1 Speech	1.00 (1.00-2.00)	1.00 (1.00-2.00)	0.00 (-1.00 to 0.00)	0.85
3.2 Facial expression	2.00 (1.00-2.00)	2.00 (1.00-2.00)	0.00 (-1.00 to 1.00)	0.95
3.3a Rigidity - neck	2.00 (1.00-2.00)	2.00 (1.00-2.00)	0.00 (-1.00 to 1.00)	0.89
3.3b Rigidity - right upper extremity	2.00 (1.00-2.00)	1.00 (1.00-2.00)	0.00 (-1.00 to 1.00)	0.86
3.3c Rigidity - left upper extremity	2.00 (1.00-3.00)	1.00 (0.00-2.00)	1.00 (0.00 to 1.00)	0.19
3.3d Rigidity - right lower extremity	1.00 (0.00-2.00)	1.00 (0.00-1.00)	0.00 (-1.00 to 1.00)	0.96
3.3e Rigidity - left lower extremity	1.00 (0.00-2.00)	0.00 (0.00-1.00)	0.00 (-1.00 to 1.00)	0.82
3.4a Finger tapping - right hand	2.00 (1.00-3.00)	2.00 (1.00-2.00)	0.00 (-1.00 to 1.00)	0.84
3.4b Finger tapping - left hand	2.00 (1.00-3.00)	2.00 (1.00-2.00)	0.00 (-1.00 to 1.00)	0.92
3.5a Hand movements - right hand	2.00 (1.00-3.00)	2.00 (1.00-2.00)	0.00 (-1.00 to 1.00)	0.83
3.5b Hand movements - left hand	1.00 (0.00-2.00)	1.00 (0.00-2.00)	0.00 (-1.00 to 1.00)	0.94
3.6a Pronation-supination - right hand	1.00 (0.00-2.00)	1.00 (0.00-2.00)	0.00 (-1.00 to 1.00)	0.97
3.6b Pronation-supination - left hand	1.00 (0.00-2.00)	1.00 (0.00-2.00)	0.00 (-1.00 to 1.00)	0.89
3.7a Toe tapping - right foot	2.00 (1.00-3.00)	2.00 (1.00-3.00)	0.00 (-1.00 to 1.00)	0.91
3.7b Toe tapping - left foot	2.00 (1.00-3.00)	2.00 (1.00-2.00)	0.00 (-1.00 to 1.00)	0.85
3.8a Leg agility - right leg	2.00 (1.00-2.00)	2.00 (1.00-2.00)	0.00 (-1.00 to 1.00)	0.97
3.8b Leg agility - left leg	1.00 (0.00-2.00)	1.00 (0.00-2.00)	0.00 (-1.00 to 1.00)	0.93
3.9 Arising from chair	1.00 (0.00-2.00)	1.00 (0.00-2.00)	0.00 (-1.00 to 1.00)	0.89
3.10 Gait	2.00 (1.00-3.00)	2.00 (1.00-3.00)	0.00 (-1.00 to 1.00)	0.91
3.11 Freezing of gait	0.00 (0.00-0.00)	0.00 (0.00-0.00)	0.00 (0.00 to 0.00)	1.00
3.12 Postural stability	1.00 (0.00-2.00)	1.00 (0.00-1.00)	0.00 (-1.00 to 1.00)	0.95
3.13 Posture	2.00 (1.00-2.00)	2.00 (1.00-2.00)	0.00 (-1.00 to 1.00)	0.98
3.14 Global spontaneity of movement	2.00 (1.00-3.00)	2.00 (1.00-3.00)	0.00 (-1.00 to 1.00)	0.89
3.15a Postural tremor - right hand	0.00 (0.00-1.00)	0.00 (0.00-1.00)	0.00 (-1.00 to 0.00)	0.91
3.15b Postural tremor - left hand	0.00 (0.00-1.00)	0.00 (0.00-1.00)	0.00 (-1.00 to 0.00)	0.91
3.16a Kinetic tremor - right hand	0.00 (0.00-1.00)	0.00 (0.00-1.00)	0.00 (-1.00 to 1.00)	0.96
3.16b Kinetic tremor - left hand	0.00 (0.00-1.00)	0.00 (0.00-1.00)	0.00 (-1.00 to 1.00)	0.92
3.17a Rest tremor amplitude - right upper extremity	0.00 (0.00-0.00)	0.00 (0.00-0.00)	0.00 (0.00 to 0.00)	1.00
3.17b Rest tremor amplitude - left upper extremity	0.00 (0.00-0.00)	0.00 (0.00-0.00)	0.00 (0.00 to 0.00)	1.00
3.17c Rest tremor amplitude - right lower extremity	0.00 (0.00-0.00)	0.00 (0.00-0.00)	0.00 (0.00 to 0.00)	1.00
3.17d Rest tremor amplitude - left lower extremity	0.00 (0.00-0.00)	0.00 (0.00-0.00)	0.00 (0.00 to 0.00)	1.00
3.17e Rest tremor amplitude - lip/jaw	0.00 (0.00-0.00)	0.00 (0.00-0.00)	0.00 (0.00 to 0.00)	1.00
3.18 Constancy of rest tremor	1.00 (0.00-1.00)	0.00 (0.00-1.00)	0.00 (-1.00 to 1.00)	0.87

Data are presented as median (IQR), as appropriate. N denotes number of participants; CI, confidence interval. MDS-UPDRS, Movement Disorder Society-Unified Parkinson's Disease Rating Scale.

Supplementary Table 12. Within-group change in MDS-UPDRS Part III subscores (items 3.1-3.18)

Item	Sham device group (n = 23) baseline	Sham device group (n = 23) follow-up	Change (95% CI)	P value	Active device group (n = 25) baseline	Active device group (n = 25) follow-up	Change (95% CI)	P value
3.1 Speech	1.00 (1.00-1.00)	1.00 (1.00-2.00)	0.00 (0.00 to 1.00)	0.31	1.00 (1.00-2.00)	1.00 (1.00-2.00)	0.00 (0.00 to 0.00)	0.85
3.2 Facial expression	2.00 (1.00-2.00)	2.00 (1.00-2.00)	0.00 (-1.00 to 1.00)	0.88	2.00 (1.00-2.00)	2.00 (1.00-2.00)	0.00 (-1.00 to 1.00)	0.95
3.3a Rigidity - neck	2.00 (2.00-3.00)	2.00 (1.00-2.00)	0.00 (-1.00 to 1.00)	0.68	2.00 (2.00-3.00)	2.00 (1.00-2.00)	0.00 (-1.00 to 1.00)	0.89
3.3b Rigidity - right upper extremity	2.00 (1.50-2.50)	2.00 (1.00-2.00)	0.00 (-1.00 to 1.00)	0.71	2.00 (2.00-2.00)	1.00 (1.00-2.00)	-1.00 (-1.00 to 0.00)	0.29
3.3c Rigidity - left upper extremity	2.00 (1.00-3.00)	2.00 (1.00-3.00)	0.00 (-1.00 to 1.00)	0.74	2.00 (0.00-3.00)	1.00 (0.00-2.00)	-1.00 (-2.00 to 0.00)	0.19
3.3d Rigidity - right lower extremity	1.00 (1.00-2.00)	1.00 (0.00-2.00)	0.00 (-1.00 to 1.00)	0.81	1.00 (1.00-2.00)	1.00 (0.00-1.00)	-1.00 (-1.00 to 0.00)	0.33
3.3e Rigidity - left lower extremity	2.00 (1.00-2.00)	1.00 (0.00-2.00)	0.00 (-1.00 to 1.00)	0.77	1.00 (0.00-2.00)	0.00 (0.00-1.00)	0.00 (-1.00 to 1.00)	0.82
3.4a Finger tapping - right hand	2.00 (2.00-3.00)	2.00 (1.00-3.00)	0.00 (-1.00 to 1.00)	0.82	2.00 (2.00-3.00)	2.00 (1.00-2.00)	0.00 (-1.00 to 1.00)	0.84
3.4b Finger tapping - left hand	2.00 (1.00-2.00)	2.00 (1.00-3.00)	0.00 (-1.00 to 1.00)	0.85	2.00 (1.00-3.00)	2.00 (1.00-2.00)	0.00 (-1.00 to 1.00)	0.92
3.5a Hand movements - right hand	2.00 (1.00-3.00)	2.00 (1.00-3.00)	0.00 (-1.00 to 1.00)	0.87	2.00 (1.00-3.00)	2.00 (1.00-2.00)	0.00 (-1.00 to 1.00)	0.83
3.5b Hand movements - left hand	2.00 (1.00-2.00)	1.00 (0.00-2.00)	0.00 (-1.00 to 1.00)	0.76	2.00 (1.00-2.00)	1.00 (0.00-2.00)	0.00 (-1.00 to 1.00)	0.94
3.6a Pronation-supination - right hand	2.00 (1.00-2.00)	1.00 (0.00-2.00)	0.00 (-1.00 to 1.00)	0.80	2.00 (1.00-2.00)	1.00 (0.00-2.00)	0.00 (-1.00 to 1.00)	0.97
3.6b Pronation-supination - left hand	2.00 (1.00-2.00)	1.00 (0.00-2.00)	0.00 (-1.00 to 1.00)	0.83	2.00 (1.00-2.00)	1.00 (0.00-2.00)	0.00 (-1.00 to 1.00)	0.89
3.7a Toe tapping - right foot	2.00 (1.00-3.00)	2.00 (1.00-3.00)	0.00 (-1.00 to 1.00)	0.84	2.00 (1.00-3.00)	2.00 (1.00-3.00)	0.00 (-1.00 to 1.00)	0.90
3.7b Toe tapping - left foot	2.00 (1.00-3.00)	2.00 (1.00-2.00)	0.00 (-1.00 to 1.00)	0.86	2.00 (1.00-2.00)	2.00 (1.00-2.00)	0.00 (-1.00 to 1.00)	0.85
3.8a Leg agility - right leg	2.00 (1.00-2.00)	2.00 (1.00-2.00)	0.00 (-1.00 to 1.00)	0.88	2.00 (1.00-3.00)	2.00 (1.00-2.00)	0.00 (-1.00 to 1.00)	0.97
3.8b Leg agility - left leg	1.00 (0.00-2.00)	1.00 (0.00-2.00)	0.00 (-1.00 to 1.00)	0.91	1.00 (0.00-2.00)	1.00 (0.00-2.00)	0.00 (-1.00 to 1.00)	0.93

3.9 Arising from chair	2.00 (1.00-2.00)	1.00 (0.00-2.00)	0.00 (-1.00 to 1.00)	0.75	2.00 (1.00-2.00)	1.00 (0.00-2.00)	0.00 (-1.00 to 1.00)	0.89
3.10 Gait	2.00 (1.00-2.00)	2.00 (1.00-3.00)	0.00 (-1.00 to 1.00)	0.78	2.00 (1.00-3.00)	2.00 (1.00-3.00)	0.00 (-1.00 to 1.00)	0.91
3.11 Freezing of gait	0.00 (0.00-0.00)	0.00 (0.00-0.00)	0.00 (0.00 to 0.00)	1.00	0.00 (0.00-0.00)	0.00 (0.00-0.00)	0.00 (0.00 to 0.00)	1.00
3.12 Postural stability	1.00 (0.00-2.00)	1.00 (0.00-2.00)	0.00 (-1.00 to 1.00)	0.80	1.00 (0.00-2.00)	1.00 (0.00-1.00)	0.00 (-1.00 to 1.00)	0.95
3.13 Posture	2.00 (1.00-2.00)	2.00 (1.00-2.00)	0.00 (-1.00 to 1.00)	0.81	2.00 (1.00-2.00)	2.00 (1.00-2.00)	0.00 (-1.00 to 1.00)	0.98
3.14 Global spontaneity of movement	2.00 (1.00-2.00)	2.00 (1.00-3.00)	0.00 (-1.00 to 1.00)	0.87	2.00 (1.00-3.00)	2.00 (1.00-3.00)	0.00 (-1.00 to 1.00)	0.89
3.15a Postural tremor - right hand	0.00 (0.00-1.00)	0.00 (0.00-1.00)	0.00 (-1.00 to 0.00)	0.94	0.00 (0.00-1.00)	0.00 (0.00-1.00)	0.00 (-1.00 to 0.00)	0.91
3.15b Postural tremor - left hand	0.00 (0.00-1.00)	0.00 (0.00-1.00)	0.00 (-1.00 to 1.00)	0.92	0.00 (0.00-1.00)	0.00 (0.00-1.00)	0.00 (-1.00 to 0.00)	0.91
3.16a Kinetic tremor - right hand	0.00 (0.00-1.00)	0.00 (0.00-1.00)	0.00 (-1.00 to 1.00)	0.91	0.00 (0.00-1.00)	0.00 (0.00-1.00)	0.00 (-1.00 to 1.00)	0.96
3.16b Kinetic tremor - left hand	0.00 (0.00-1.00)	0.00 (0.00-1.00)	0.00 (-1.00 to 1.00)	0.91	0.00 (0.00-1.00)	0.00 (0.00-1.00)	0.00 (-1.00 to 1.00)	0.92
3.17a Rest tremor amplitude - right upper extremity	0.00 (0.00-0.00)	0.00 (0.00-0.00)	0.00 (0.00 to 0.00)	1.00	0.00 (0.00-0.00)	0.00 (0.00-0.00)	0.00 (0.00 to 0.00)	1.00
3.17b Rest tremor amplitude - left upper extremity	0.00 (0.00-0.00)	0.00 (0.00-0.00)	0.00 (0.00 to 0.00)	1.00	0.00 (0.00-0.00)	0.00 (0.00-0.00)	0.00 (0.00 to 0.00)	1.00
3.17c Rest tremor amplitude - right lower extremity	0.00 (0.00-0.00)	0.00 (0.00-0.00)	0.00 (0.00 to 0.00)	1.00	0.00 (0.00-0.00)	0.00 (0.00-0.00)	0.00 (0.00 to 0.00)	1.00
3.17d Rest tremor amplitude - left lower extremity	0.00 (0.00-0.00)	0.00 (0.00-0.00)	0.00 (0.00 to 0.00)	1.00	0.00 (0.00-0.00)	0.00 (0.00-0.00)	0.00 (0.00 to 0.00)	1.00
3.17e Rest tremor amplitude - lip/jaw	0.00 (0.00-0.00)	0.00 (0.00-0.00)	0.00 (0.00 to 0.00)	1.00	0.00 (0.00-0.00)	0.00 (0.00-0.00)	0.00 (0.00 to 0.00)	1.00
3.18 Constancy of rest tremor	1.00 (0.00-1.00)	1.00 (0.00-1.00)	0.00 (-1.00 to 1.00)	0.88	1.00 (0.00-1.00)	0.00 (0.00-1.00)	0.00 (-1.00 to 1.00)	0.87

Data are presented as median (IQR), as appropriate. N denotes number of participants; CI, confidence interval. MDS-UPDRS, Movement Disorder Society-Unified Parkinson's Disease Rating Scale.

Supplementary Table 13. Within group difference in outcomes at follow up

Measure	Sham device group (n = 23) baseline	Sham device group (n = 23) follow-up	Change (95% CI)	P value	Active device group (n = 25) baseline	Active device group (n = 25) follow-up	Change (95% CI)	P value
MDS-UPDRS Part I	14.0 (10.0-21.0)	13.0 (10.5-17.5)	0.0 (0.0 to 3.0)	0.41	22.0 (14.0-26.0)	15.0 (11.0-24.0)	4.0 (2.0 to 7.0)	<0.001
MDS-UPDRS Part II	16.0 (9.0-21.5)	15.0 (7.0-22.5)	0.0 (2.0 to 3.0)	0.98	21.0 (11.0-25.0)	12.0 (9.0-19.0)	-4.0 (-7.0 to -1.0)	<0.001
MDS-UPDRS Part IV	8.7 (5.3)	8.0 (5.0-11.0)	0.0 (0.0 to 1.0)	0.85	10.0 (4.6)	5.0 (4.0-9.0)	-4.0 (-5.0 to -3.0)	<0.001
Functional Gait Assessment	17.0 (14.5-18.5)	17.6 (4.2)	1.2 (0.0 to 2.5)	0.05	18.0 (14.0-20.0)	19.0 (16.0-22.0)	3.0 (2.0 to 4.0)	<0.001
Timed Up and Go, single task	10.4 (8.4-13.1)	10.7 (3.6)	0.0 (-1.3 to 1.3)	0.99	10.0 (8.2-12.1)	9.1 (8.4-10.8)	-1.1 (-4.7 to 0.3)	0.03
Timed Up and Go, dual task	14.2 (11.1-20.1)	13.3 (9.7-18.5)	0.0 (-2.8 to 0.8)	0.26	13.2 (10.2-19.1)	12.1 (10.6-17.5)	-1.8 (-7.2 to 1.7)	0.03
BRAIN tap KS (most affected side)	34.5 (31.0-46.8)	38.0 (36.0-47.0)	3.0 (0.0 to 7.0)	0.06	40.0 (37.0-46.0)	47.0 (40.0-55.0)	3.0 (0.0 to 12.0)	0.007
Digital finger tapping KS (most affected side	67.5 (54.0-73.0)	63.5 (49.0-80.0)	-1.3 (-8.7 to 6.0)	0.79	64.0 (54.0-80.0)	76.0 (54.0-87.0)	16.2 (-9.8 to 42.2)	0.10
Activities-specific Balance Confidence	53.8 (41.9-87.8)	62.5 (24.8)	0.0 (-9.4 to 6.3)	0.94	55.0 (41.3-80.0)	56.3 (47.5-86.3)	3.8 (-1.9 to 15.6)	0.01
Pittsburgh Sleep Quality Index	10.9 (5.4)	10.2 (4.9)	-0.7 (-2.2 to 0.9)	0.41	10.9 (5.0)	8.0 (5.0-13.0)	-2.0 (-3.0 to -1.0)	<0.001
PDQ-39 Summary Index	25.9 (11.5-43.4)	27.8 (11.9-42.0)	0.1 (-2.9 to 3.1)	0.93	35.7 (27.9-51.1)	29.1 (17.7-40.4)	-7.5 (-11.5 to -3.4)	<0.001
PDQ-39 Mobility	40.0 (6.3-63.0)	32.5 (8.8-61.3)	0.0 (0.0 to 5.0)	0.65	40.0 (22.5-65.0)	25.0 (10.0-57.5)	-7.5 (-15.0 to 0.0)	0.02
PDQ-39 Activities of daily living	33.3 (8.3-47.9)	29.2 (4.2-45.8)	-4.2 (-8.3 to 0.0)	0.07	37.5 (16.7-62.5)	20.8 (4.2-50.0)	-12.5 (-12.5 to -4.2)	<0.001
PDQ-39 Emotional wellbeing	20.8 (8.3-37.5)	20.8 (12.5-56.3)	4.2 (-4.2 to 4.2)	0.33	41.7 (25.0-75.0)	37.5 (12.5-50.0)	-4.2 (-12.5 to 0.0)	0.009
PDQ-39 Stigma	12.5 (6.3-40.6)	12.5 (0.0-46.9)	0.0 (0.0 to 0.0)	0.91	25.0 (6.3-56.3)	18.8 (6.3-37.5)	-6.3 (-18.8 to 0.0)	0.03
PDQ-39 Social support	0.0 (0.0-16.7)	8.3 (0.0-20.8)	0.0 (0.0 to 0.0)	0.11	0.0 (25.0-25.0)	8.3 (0.0-25.0)	0.0 (0.0 to 8.3)	0.47
PDQ-39 Cognition	25.0 (15.6-40.6)	25.0 (18.8-50.0)	0.0 (-6.3 to 0.0)	1.00	37.5 (25.0-56.3)	25.0 (12.5-37.5)	-12.5 (-25.0 to 0.0)	0.004

PDQ-39 Communication	16.7 (0.0-41.7)	16.7 (4.2-37.5)	0.0 (0.0 to 0.0)	0.50	33.3 (16.7-50.0)	16.7 (8.3-33.3)	-8.3 (-16.7 to 0.0)	0.007
PDQ-39 Bodily discomfort	50.0 (25.0-58.3)	33.3 (20.8-58.3)	0.0 (-8.3 to 0.2)	0.28	33.3 (16.7-66.7)	33.3 (8.3-66.7)	0.0 (-8.3 to 8.3)	0.96
HADS Anxiety	3.5 (3.0-9.5)	5.0 (3.0-9.0)	-1.0 (-1.2 to 1.1)	0.86	11.0 (7.0-13.0)	10.0 (7.0-12.0)	-9.6 (-2.1 to 0.2)	0.05
HADS Depression	6.0 (4.0-10.0)	6.0 (4.0-9.5)	0.0 (-1.0 to 0.0)	0.88	8.0 (6.0-10.0)	8.0 (5.0-11.0)	0.0 (-2.0 to 1.0)	0.24
Fatigue Severity Scale	36.0 (22.0-46.5)	40.0 (19.5-46.0)	1.0 (0.0 to 3.0)	0.38	38.0 (30.0-49.0)	36.0 (32.0-44.0)	-3.0 (-10.0 to 0.0)	0.23

Data are presented as median (IQR) or mean (SD), as appropriate. N denotes number; CI, confidence interval. MDS-UPDRS, Movement Disorder Society-Unified Parkinson's Disease Rating Scale; FGA, Functional Gait Assessment; TUG, Timed Up and Go; BRAIN tap, Bradykinesia Akinesia Incoordination tap test; KS, Kinesia Score; DFT, Digital Finger Tapping; ABC, Activities-specific Balance Confidence; PSQI, Pittsburgh Sleep Quality Index; PDQ-39, Parkinson's Disease Questionnaire-39; HADS, Hospital Anxiety and Depression Scale; FSS, Fatigue Severity Scale.
